# Dynamic Cerebral Autoregulation Reproducibility Is Affected by Physiological Variability

**DOI:** 10.3389/fphys.2019.00865

**Published:** 2019-07-09

**Authors:** Marit L. Sanders, Jan Willem J. Elting, Ronney B. Panerai, Marcel Aries, Edson Bor-Seng-Shu, Alexander Caicedo, Max Chacon, Erik D. Gommer, Sabine Van Huffel, José L. Jara, Kyriaki Kostoglou, Adam Mahdi, Vasilis Z. Marmarelis, Georgios D. Mitsis, Martin Müller, Dragana Nikolic, Ricardo C. Nogueira, Stephen J. Payne, Corina Puppo, Dae C. Shin, David M. Simpson, Takashi Tarumi, Bernardo Yelicich, Rong Zhang, Jurgen A. H. R. Claassen

**Affiliations:** ^1^Department of Geriatric Medicine, Radboudumc Alzheimer Center, Donders Institute for Brain, Cognition and Behaviour, Radboud University Medical Center, Nijmegen, Netherlands; ^2^Department of Neurology, University Medical Center Groningen, Groningen, Netherlands; ^3^Department of Cardiovascular Sciences, NIHR Leicester Biomedical Research Centre, Glenfield Hospital, Leicester, United Kingdom; ^4^Department of Intensive Care, University of Maastricht, Maastricht University Medical Center, Maastricht, Netherlands; ^5^Department of Neurology, Faculty of Medicine, Hospital das Clinicas University of São Paulo, São Paulo, Brazil; ^6^Department of Applied Mathematics and Computer Science, Faculty of Natural Sciences and Mathematics, Universidad del Rosario, Bogotá, Colombia; ^7^Department of Engineering Informatics, Institute of Biomedical Engineering, University of Santiago, Santiago, Chile; ^8^Department of Clinical Neurophysiology, Maastricht University Medical Centre, Maastricht, Netherlands; ^9^Department of Electronic Engineering (ESAT), Stadius Center for Dynamical Systems, Signal Processing and Data Analytics, Katholieke Universiteit Leuven, Leuven, Belgium; ^10^Interuniversity Microelectronics Centre, Leuven, Belgium; ^11^Department of Electrical, Computer and Software Engineering, McGill University, Montreal, QC, Canada; ^12^Department of Engineering Science, University of Oxford, Oxford, United Kingdom; ^13^Department of Biomedical Engineering, University of Southern California, Los Angeles, CA, United States; ^14^Department of Bioengineering, McGill University, Montreal, QC, Canada; ^15^Department of Neurology, Luzerner Kantonsspital, Luzern, Switzerland; ^16^Faculty of Engineering and the Environment, Institute of Sound and Vibration Research, University of Southampton, Southampton, United Kingdom; ^17^Departamento de Emergencia, Hospital de Clínicas, Universidad de la República, Montevideo, Uruguay; ^18^Institute for Exercise and Environmental Medicine, Presbyterian Hospital of Dallas, University of Texas Southwestern Medical Center, Dallas, TX, United States

**Keywords:** ARI index, cerebral blood flow, cerebral hemodynamics, transcranial Doppler, transfer function analysis

## Abstract

Parameters describing dynamic cerebral autoregulation (DCA) have limited reproducibility. In an international, multi-center study, we evaluated the influence of multiple analytical methods on the reproducibility of DCA. Fourteen participating centers analyzed repeated measurements from 75 healthy subjects, consisting of 5 min of spontaneous fluctuations in blood pressure and cerebral blood flow velocity signals, based on their usual methods of analysis. DCA methods were grouped into three broad categories, depending on output types: (1) transfer function analysis (TFA); (2) autoregulation index (ARI); and (3) correlation coefficient. Only TFA gain in the low frequency (LF) band showed good reproducibility in approximately half of the estimates of gain, defined as an intraclass correlation coefficient (ICC) of >0.6. None of the other DCA metrics had good reproducibility. For TFA-like and ARI-like methods, ICCs were lower than values obtained with surrogate data (*p* < 0.05). For TFA-like methods, ICCs were lower for the very LF band (gain 0.38 ± 0.057, phase 0.17 ± 0.13) than for LF band (gain 0.59 ± 0.078, phase 0.39 ± 0.11, *p* ≤ 0.001 for both gain and phase). For ARI-like methods, the mean ICC was 0.30 ± 0.12 and for the correlation methods 0.24 ± 0.23. Based on comparisons with ICC estimates obtained from surrogate data, we conclude that physiological variability or non-stationarity is likely to be the main reason for the poor reproducibility of DCA parameters.

## Introduction

The importance of cerebral autoregulation (CA) has been clearly established, as a cerebro-protective mechanism to alterations in blood pressure (BP) by keeping cerebral blood flow (CBF) relatively constant (van Beek et al., 2008). Dynamic CA (DCA) is the transient cerebrovascular response to rapid changes in BP (Aaslid et al., 1989). Compared to the more classical modality of “static” autoregulation, that often requires the use of pharmacological agents to induce steady-state changes in BP (Tiecks et al., 1995), DCA has benefitted from recent developments in non-invasive techniques to record CBF and BP, and it is now the preferred approach for assessment of CA in physiological and clinical studies.

Despite its many advantages, protocols to reliably assess DCA remain the object of considerable debate (Simpson and Claassen, 2018a,b; Tzeng and Panerai, 2018a,b). On the one hand, maneuvers that induce relatively large and rapid changes in BP, such as the sudden release of compressed thigh cuffs (Aaslid et al., 1989), lead to recordings with better signal-to-noise ratio and the possibility of visualizing and quantifying the DCA response with measurements as short as 30 s. On the other hand, using the spontaneous fluctuations in BP and CBF, that can be observed in most individuals, allows estimation of DCA parameters at rest, without the need for a physiological disturbance or challenge. This can lead to better acceptance and feasibility in most clinical conditions.

Which road to take? The answer to this fundamental question is not straightforward as it is unlikely that a single protocol will be suitable for all different scenarios of patient care and physiological intervention (Simpson and Claassen, 2018a,b; Tzeng and Panerai, 2018a,b).

A definition of an optimal protocol could be one which, combined with robust modeling techniques (Panerai, 2008), leads to the best sensitivity and specificity performance for detection of CA disturbances, as well as predictive accuracy for patient prognosis.

Before reaching this stage though, it is essential that measurement reproducibility is demonstrated as a key property of any method of assessment. This target is at the forefront of the collaborative initiatives promulgated by the International Cerebral Autoregulation Network (CARNet) as part of the effort to identify potential sources of methodological disparity (Meel-van den Abeelen et al., 2014) and encourage technical standardization (Claassen et al., 2016). The most recent stage of this pathway is described in this article and involves an international, multi-center assessment of the reproducibility of the main parameters that are currently available to assess DCA based on spontaneous fluctuations of BP and CBF.

Examining the reproducibility of DCA parameters, obtained from spontaneous fluctuations at rest, is important due to the widespread use of this approach for both physiological and clinical studies. Early assessments of the reproducibility of the spontaneous fluctuations approach were not encouraging (Brodie et al., 2009; Gommer et al., 2010; Smirl et al., 2015), but were not regarded as the definitive answer, only as indicative of a single method, handled by a single center. This limitation was addressed in the current multi-center study. An initial report (Sanders et al., 2018) described the influence of different methods of analysis on the reproducibility of synthetic data, where surrogate time-series of CBF velocity (CBFv) were generated based on real measurements of BP, coupled with a realistic signal-to-noise ratio. These generated CBFv data were based on a linear model. Thus, compared to real CBFv data, these generated data are free of any physiological influences on the BP–CBFv relationship. Such physiological influences could include non-stationary behavior of autoregulatory function (i.e., variations in function over time), and factors known to influence CBFv (e.g., PaCO_2_, cognitive activity, autonomic nervous activity, temperature, breathing pattern).

The present communication therefore had as aim to provide a much broader description of the reproducibility of “real” estimates of DCA from 14 leading international centers, using a diversity of analytical methods. In particular, this study addressed two main objectives: (1) to compare the reproducibility of DCA parameters from these real physiological measurements to that of surrogate data and (2) to establish the influence of different analytical methods used by a variety of research centers worldwide on the reproducibility of DCA metrics.

## Materials and Methods

### Subjects

A database was created from available datasets of cerebral hemodynamic measurements from participating centers (Supplementary Table S1). Included were healthy adults >18 years of age. Exclusion criteria were uncontrolled hypertension, smoking, cardiovascular disease, diabetes, irregular heart rhythm, TIA/stroke, or significant pulmonary disease. The study has been carried out in accordance with the Code of Ethics of the World Medical Association (Declaration of Helsinki). Written informed consent was obtained from all subjects.

### Description of Datasets

Six of a total of 14 centers (Supplementary Table S1) provided datasets that consisted of two measurements from 10 to 15 healthy volunteers in each center, resulting in a total of 75 healthy subjects. Time between the two measurements varied between centers, from minutes to a maximum of 4 months. Data sets consisted of 5 min of beat-to-beat artifact free mean CBFv (transcranial Doppler ultrasound, TCD), mean BP (digital artery volume clamping), and end-tidal CO_2_ (EtCO_2_, capnography) measurements at rest. Beat-to-beat parameters were re-sampled at 10 Hz. In 22 subjects, the TCD data were unilateral. The dataset was as follows: *N* = 55 left side signals, *N* = 71 right side signals.

### DCA Analysis

Data analyses were performed by 14 participating centers. The following DCA analysis methods were used: transfer function analysis (TFA) (Panerai et al., 1998a; Zhang et al., 1998; Mitsis et al., 2002; Muller et al., 2003; Reinhard et al., 2003; Liu et al., 2005; Gommer et al., 2010; van Beek et al., 2010; Meel-van den Abeelen et al., 2014; Muller and Osterreich, 2014; Panerai, 2014), Laguerre expansion of first-order Volterra kernels or finite impulse response models (Marmarelis, 2004; Mitsis et al., 2004, 2009; Marmarelis et al., 2013, 2014a,b), wavelet analysis (Torrence and Webster, 1999; Grinsted et al., 2004; Peng et al., 2010), parametric finite-impulse response filter-based methods (Panerai et al., 2000; Simpson et al., 2001), autoregulation index (ARI) analysis (Panerai et al., 1998b), autoregressive moving average (ARMA)-based ARI methods and variant ARI methods (Panerai et al., 2003), autoregressive with exogenous input (ARX) methods (Liu and Allen, 2002; Liu et al., 2003; Panerai et al., 2003), and correlation coefficient-like indices (Heskamp et al., 2013; Caicedo et al., 2016). A summary of the methods and corresponding references are given in Table 1.

**Table 1:** Methods with corresponding output variables per center.

Center number	Method	Output variables	Category	Method group	References
1.	1.1 TFA 1.2 ARI	Coherence, Gain (cm/s/mmHg), and Phase (rad) in VLF, LF ARI	1 2	1 6	Zhang et al., 1998 Panerai et al., 1998b
2.	2.1 Laguerre expansion of first-order Volterra kernels, single input (BP) 2.2 Laguerre expansion of first-order Volterra kernels, dual input (BP, CO_2_)	Gain (cm/s/mmHg) and Phase (rad) in VLF, LF Gain (cm/s/mmHg) and Phase (rad) in VLF, LF	1 1	2 2	Marmarelis, 2004; Marmarelis et al., 2013, 2014a,b
3.	3.1 TFA 3.2 TFA	Coherence, Gain (cm/s/mmHg), Phase (rad) in VLF, LF Coherence, Gain (%/%) in VLF, LF	1 1	1 1	Zhang et al., 1998
4.	4.1 ARI (FFT) 4.2 ARI (moving average 1) 4.3 ARI (moving average 2)	ARI ARI ARI	2 2 2	6 7 7	Panerai et al., 1998b, 2003
5.	5.1 TFA 5.2 Oblique and orthogonal subspace projections	Coherence, Gain (cm/s/mmHg), Phase (rad) in VLF, LF Subspace ratio’s	1 3	1 10	Zhang et al., 1998 Caicedo et al., 2016
6.	6.1 TFA	Coherence, Gain (cm/s/mmHg), Phase (rad) in VLF, LF	1	1	Muller et al., 2003; Muller and Osterreich, 2014
7	7.2 TFA	Coherence, Gain (cm/s/mmHg), Phase (rad) in VLF, LF	1	1	Gommer et al., 2010
8	8.1 ARX 8.2 Wavelet analysis	ARX coefficient (third) Synchronization index, Phase (rad) in VLF, LF	2 1	7 3	Liu and Allen, 2002; Liu et al., 2003; Panerai et al., 2003 Peng et al., 2010
9.	9.1 TFA 9.2 Convergent cross mapping	Coherence, Gain (cm/s/mmHg), Phase (rad) in VLF, LF CCM correlation coefficient	1 3	1 10	van Beek et al., 2010, 2012 Heskamp et al., 2013
11.	11.1 TFA 11.2 TFA 11.3 TFA 11.4 Univariate TFA (parametric method) 11.5 Univariate impulse response (parametric method) 11.6 Multivariate TFA (parametric method)	Coherence, Gain (cm/s/mmHg), Phase (rad) in VLF, LF Coherence, Gain (%/mmHg), Phase (rad) in VLF, LF Coherence, Gain (%/%) in VLF, LF Coherence, Gain (%/%), Phase (rad) in LF The second filter coefficient (h_1_) of the estimated FIR Gain (%/%) and Phase (rad) for LF band	1 1 1 1 2 1	1 1 1 4 9 4	Panerai et al., 2000; Simpson et al., 2001
12.	12.1 TFA 12.2 ARI 12.3 Wavelet coherence analysis	Coherence, Gain (cm/s/mmHg), Phase (rad) in VLF, LF ARI Gain (cm/s/mmHg) and Phase (rad) in VLF, LF	1 2 1	1 6 3	Zhang et al., 1998 Panerai et al., 1998b Torrence and Webster, 1999; Grinsted et al., 2004
13.	13.1 TFA	Coherence, Gain (cm/s/mmHg), Phase (rad) in VLF, LF	1	1	Panerai et al., 1998a
14.	14.1 ARX models: 1 input 14.2 ARX models: 2 inputs 14.3 Laguerre expansion FIR models, single input (BP) 14.4 Laguerre expansion FIR models, dual input (BP, CO_2_) 14.5 TFA	Gain (cm/s/mmHg), Phase (rad) in VLF, LF Gain (cm/s/mmHg), Phase (rad) in VLF, LF Gain (cm/s/mmHg), Phase (rad) in VLF, LF Gain (cm/s/mmHg), Phase (rad) in VLF, LF Coherence, Gain (cm/s/mmHg), Phase (rad) in VLF, LF	1 1 1 1 1	5 5 2 2 1	Mitsis et al., 2002, 2009 Mitsis et al., 2004; Kostoglou et al., 2014 Meel-van den Abeelen et al., 2014

*Category: 1, TFA-like methods; 2, ARI-like methods; 3, correlation-like methods. Method group: 1, TFA; 2, Laguerre expansions; 3, Wavelets; 4, IR-filter; 5, ARX; 6, ARI; 7, ARMA-ARI/ARX; 9, IR-filter; 10, correlation coefficient; VLF, very low frequency; LF, low frequency; BP, blood pressure; FFT, fast Fourier transform; ARI, autoregulation index; ARX, autoregressive model with exogenous input. Center names are listed in Supplementary Table S1, individual method settings are listed in Supplementary Table S4.*

### Reproducibility of DCA Metrics

For the reproducibility and variability analysis of the DCA parameters, DCA methods were grouped into three broad categories: (1) TFA-like output, (2) ARI-like output; and (3) correlation coefficient-like outputs. These categories were created from the perspective of similar output parameters, not because of similarity on mathematical grounds. In general, all centers were free to use their own settings to cover the standard frequency range between 0 and 0.5 Hz. In the majority of cases though, for the TFA-like output methods, the settings for TFA were similar to what was later proposed in the CARNet White Paper (Claassen et al., 2016). In summary, this involved spectral estimates using the Welch method with multiple segments of data of at least 100 s, 50% superposition, and cosine windowing to reduce spectral leakage. Individual method settings are listed in Supplementary Table S4. Estimates of gain and phase were averaged for different frequency bands, very low frequency (VLF), and LF bands (Supplementary Table S4; Claassen et al., 2016).

The ARI-like output methods consisted of time domain estimates of the impulse or step response, using the inverse Fourier transform of gain and phase, or ARMA models (Panerai et al., 1998b, 2003; Liu and Allen, 2002; Liu et al., 2003).

Finally, the correlation coefficient-like outputs consisted of a single parameter, obtained by linear regression or similar methods (Heskamp et al., 2013; Caicedo et al., 2016).

### Statistical Analysis

We assessed reproducibility as follows: To quantify the level of agreement between first and second measurement, we applied the Bland–Altman method to obtain mean difference (or bias) and to determine limits of agreement (LOA). This was done for the methods in the TFA-like, ARI-like, and correlation-like category. A non-parametric Wilcoxon signed rank test was used to check if there were significant differences between left and right side results. Left and right output results were averaged for further analyses. To correct for abnormal data distributions, Box–Cox transformations were performed, which is a power transformation with different power levels (Box and Cox, 1964). Within one analysis method, the same transformation was applied to both the first and second measurement, but different transformations may be used for different methods and different variables.

Further quantification of agreement between the repeated measurements for all DCA analysis methods was determined by one-way intraclass correlation coefficient (ICC) analysis. ICC results of TFA-like methods combined for the parameters gain and phase were compared for VLF and LF. Furthermore, the differences between the ICC results of previously obtained surrogate data (Sanders et al., 2018) and physiological data were analyzed for the methods combined in parameters gain VLF, gain LF, phase VLF, phase LF, ARI, and correlation. These differences between ICC parameter values were tested with the paired Wilcoxon signed rank test, considering that most parameters, such as TFA estimates, are not normally distributed. SPSS 22 was used for all analyses; a value of *p* < 0.05 was adopted to indicate statistical significance.

Interpretation of the absolute and maximal values of ICC were based on often quoted guidelines: poor (ICC < 0.40); fair (0.40–0.59; good (0.60–0.74); and excellent (0.75–1.00) (Cicchetti, 1994).

## Results

Subject characteristics are listed in Table 2. No significant differences were found for MAP, CBFv, and EtCO_2_ for the two measurements (T1 and T2).

**Table 2:** Subject characteristics and hemodynamic parameters.

*N*	75	
Age (years)	47.8 ± 18.6	
Female [*n* (%)]	33 (44)	
Use of AHD [*n* (%)]	5 (6.7)	
Use of NSAID [*n* (%)]	4 (5.3)	
MCI [*n* (%)]	4 (5.3)	

	**T1**	**T2**

MAP (mmHg)	90.1 ± 14.9	87.6 ± 14.8
MCBFv (cm/s)	56.3 ± 13.4	56.2 ± 12.5
EtCO_2_ (kPA)	5.0 ± 0.5	5.0 ± 0.5

*Values are presented as mean ± SD or *n* (%). AHD, antihypertensive drugs; NSAID, nonsteroidal anti-inflammatory drug; MCI, mild cognitive impairment; MAP, mean arterial pressure; MCBFv, mean blood flow velocity; EtCO_*2*_, end-tidal CO_*2*_ of measurement 1 (T1) and measurement 2 (T2).*

**FIGURE 1:**
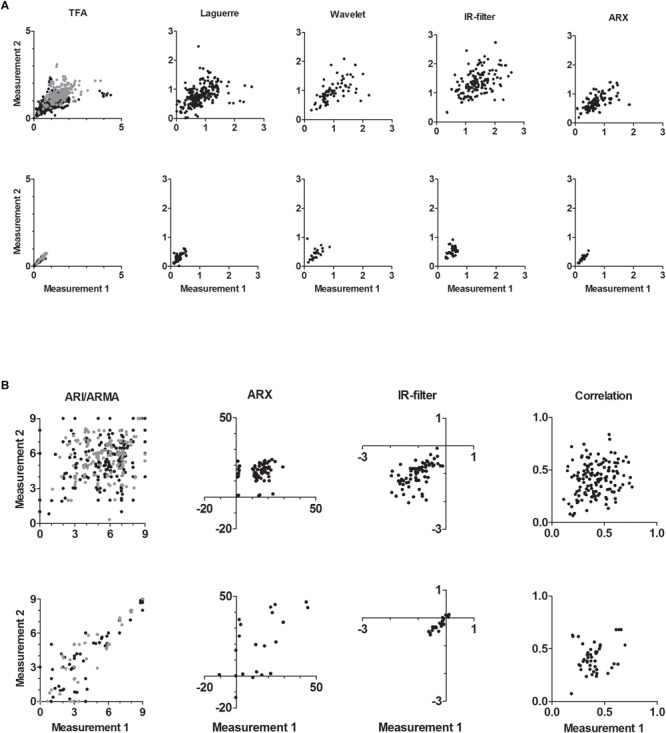
**(A)** Gain LF results of TFA-like methods for repeated measurements. Top row: physiological data, bottom row: surrogate data. For each method group (TFA, Laguerre, Wavelet, IR-filter, and ARX) the results of similar methods are combined (Table 1). TFA: black dots are 10 methods (cm/s/mmHg), gray dots are 3 methods (%/% or %/mmHg); Laguerre: 4 methods (cm/s/mmHg); Wavelet: 1 method (cm/s/mmHg); IR-filter: 2 methods (%/%); ARX: 2 methods (cm/s/mmHg). See Supplementary Figures S1–S3 for Phase VLF/LF and Gain VLF. **(B)** ARI-like results of different methods for repeated measurements. Top row: physiological data, bottom row: surrogate data. For each method group (ARI/ARMA, ARX, IR-filter, and correlation) the results of similar methods are combined (Table 1). ARI: black dots are three methods (ARI 0–9 arbitrary units); gray dots are two methods (ARMA-ARI 0–9 arbitrary units); ARX: one method (ARX coefficient); IR-filter: one method (arbitrary units); correlation: two methods.

The scatterplots of Figure 1A show examples of TFA-like metrics of the estimated LF gain and Figure 1B of the ARI-like results of the repeated measurements for both physiological and surrogate data. The figures show a difference in distribution of the data between Figure 1A,B, with a higher correlation between the repeated measurements for lower gain values only in the TFA-like results. Despite the lower number of cases in the surrogate results, it is clearly shown that there is less variability in the surrogate data (bottom) compared to physiological data (top) for all TFA-like methods (Figure 1A) and the ARI an IR-filter methods (Figure 1B). Physiological data are presented in Supplementary Tables S2a,k.

**FIGURE 2:**
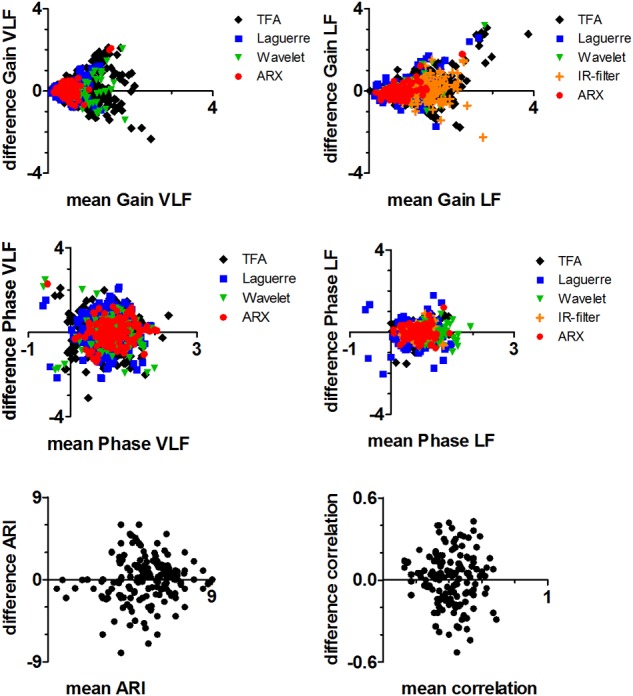
Bland–Altman plot of TFA-like parameters: gain VLF (top left), gain LF (top right), phase VLF (middle left), and phase LF (middle right); ARI-like parameters (bottom left); correlation-like parameters (bottom right). Units are similar to Figure 1A,B.

Comparing different autoregulation metrics with Bland–Altman analysis, we see a difference between gain variables and all the other variables (Figure 2). Both gain VLF and LF show a strong increase in the difference between two measurements on the *y*-axis for higher values of mean gain on the *x*-axis. For the smallest values of gain, where the DCA is considered most effective, the agreement is the strongest. Results for T1, T2, bias (T1-T2), and the LOA of the different method categories per method group are listed in Table 3. Each method group corresponds to results of several methods combined (Table 1 and Supplementary Tables S3a–c).

**Table 3:** Bland–Altman results for each method subcategory and variable.

				Left						Right			
		
Method groups	Variable	T1	T2	Bias	INT	LLOA	ULOA	T1	T2	Bias	INT	LLOA	ULOA
**TFA-like**													
TFA	Gain VLF	0.68±0.43	0.59±0.30	0.09±0.40	0.78	–0.69	0.87	0.68±0.46	0.60±0.31	0.07±0.42	0.82	–0.75	0.88
	Gain LF	1.02±0.58	0.94±0.46	0.08±0.45	0.89	–0.81	0.97	1.02±0.66	0.92±0.46	0.10±0.50	0.98	–0.88	1.08
	Phase VLF	0.87±0.44	0.86±0.50	0.01±0.58	1.14	–1.13	1.15	0.87±0.46	0.89±0.52	–0.02±0.65	1.27	–1.29	1.25
	Phase LF	0.68±0.25	0.69±0.23	–0.01±0.24	0.46	–0.47	0.45	0.69±0.27	0.69±0.24	0.01±0.26	0.52	–0.51	0.52
Laguerre	Gain VLF	0.50±0.29	0.43±0.18	0.07±0.29	0.57	–0.50	0.65	0.49±0.29	0.43±0.19	0.06±0.27	0.54	–0.48	0.60
	Gain LF	0.86±0.44	0.77±0.31	0.09±0.40	0.78	–0.69	0.88	0.86±0.51	0.77±0.30	0.10±0.42	0.83	–0.74	0.93
	Phase VLF	0.81±0.51	0.89±0.51	–0.08±0.70	1.37	–1.45	1.29	0.81±0.52	0.94±0.52	–0.13±0.72	1.42	–1.55	1.29
	Phase LF	0.65±0.30	0.65±0.31	0.00±0.39	0.77	–0.77	0.77	0.64±0.32	0.69±0.34	–0.05±0.41	0.79	–0.84	0.75
Wavelet	Gain VLF	0.91±0.47	0.79±0.36	0.11±0.54	1.05	–0.94	1.16	0.89±0.49	0.83±0.36	0.05±0.53	1.04	–1.00	1.09
	Gain LF	1.04±0.51	0.97±0.37	0.08±0.47	0.93	–0.85	1.00	1.06±0.63	0.95±0.37	0.11±0.55	1.08	–0.97	1.19
	Phase VLF	0.89±0.62	1.05±0.55	–0.12±0.70	1.38	–1.49	1.26	0.96±0.49	1.06±0.66	–0.10±0.70	1.36	–1.46	1.27
	Phase LF	0.91±0.32	0.95±0.30	–0.05±0.30	0.58	–0.63	0.54	0.94±0.31	0.97±0.32	–0.03±0.30	0.58	–0.61	0.55
IR-filter	Gain LF	1.46±0.55	1.28±0.40	0.18±0.18	0.35	–0.17	0.52	1.40±0.55	1.27±0.40	0.12±0.46	0.90	–0.77	1.02
	Phase LF	0.59±0.20	0.63±0.18	–0.04±-0.04	–0.08	0.04	–0.12	0.61±0.21	0.63±0.23	–0.02±0.25	0.50	–0.52	0.47
ARX	Gain VLF	0.48±0.29	0.42±0.17	0.06±0.29	0.58	–0.52	0.64	0.48±0.36	0.42±0.18	0.06±0.34	0.67	–0.61	0.74
	Gain LF	0.81±0.39	0.74±0.27	0.07±0.30	0.58	–0.51	0.66	0.81±0.50	0.73±0.27	0.08±0.38	0.74	–0.65	0.82
	Phase VLF	1.05±0.47	1.07±0.43	–0.02±0.50	0.99	–1.01	0.97	1.07±0.47	1.05±0.50	0.01±0.58	1.14	–1.13	1.16
	Phase LF	0.73±0.30	0.74±0.25	–0.01±0.32	0.62	–0.63	0.61	0.73±0.32	0.73±0.26	0.00±0.32	0.62	–0.62	0.62
**ARI-like**													
ARI		5.48±1.92	5.74±1.62	–0.26±2.12	4.15	–4.40	3.89	5.72±1.89	5.74±1.58	–0.03±2.36	4.63	–4.66	4.60
ARMA-ARI/ARX		8.38±5.32	8.38±5.30	0.00±4.74	9.30	–9.30	9.30	8.27±5.91	9.15±5.92	–0.88±4.49	8.80	–9.68	7.92
IR-filter		–1.07±0.56	–1.02±0.47	–0.06±0.56	1.10	–1.15	1.04	–1.06±0.53	–0.99±0.42	–0.07±0.50	0.98	–1.05	0.92
Correlation-like													
Correlation		0.45±0.14	0.42±0.15	0.03±0.19	0.37	–0.33	0.40	0.44±0.14	0.42±0.15	0.02±0.19	0.38	–0.35	0.40

*For each method group the results of similar methods are combined. Methods and units are listed in Table 1. T1, measurement 1; T2, measurement 2; bias, T1-T2; INT, interval (=1.96 ^∗^*SD*_bias_); LLOA, upper limit of agreement (=mean_bias_-interval); ULOA, lower limit of agreement (=mean_bias_+interval); TFA, transfer function analysis; IR-filter, impulse response filter; ARX, autoregressive model with exogenous input; ARI, autoregulation index; VLF, very low frequency; LF, low frequency.*

**FIGURE 3:**
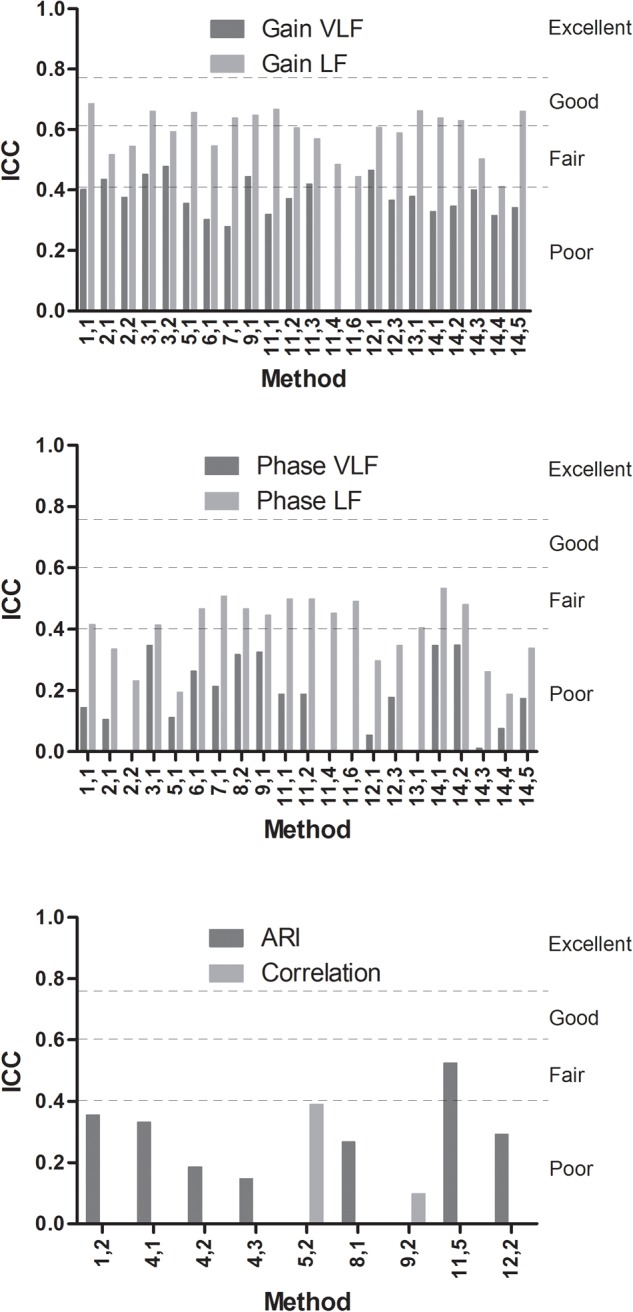
ICC values for methods using TFA or similar approaches with gain VLF and LF (top), phase VLF or LF (middle), and ARI or correlation-like methods (bottom). Results are shown per method (Table 1). ICC values <0.40: poor, between 0.40 and 0.59: fair, between 0.60 and 0.74: good, and between 0.75 and 1.00: excellent (Cicchetti, 1994).

Left and right ICC results were not different. ICC analysis of physiological data is shown in Figure 3. Despite minor differences in ICC values between methods, 12 methods qualified as having good reproducibility (ICC > 0.6). TFA-like and ARI-like methods scored significantly higher ICC for surrogate data compared to physiological data, combined for centers using the same methods, for gain VLF (*p* < 0.001), gain LF (*p* < 0.001), phase VLF (*p* < 0.001), phase LF (*p* < 0.001), and ARI (*p* = 0.018) (Sanders et al., 2018). Only the correlation-like methods did not score higher ICC values for surrogate data compared to physiological data (*p* = 0.18). ICC results of the surrogate data are presented in Supplementary Tables S5a,b.

For the TFA-like methods, ICC gain VLF [mean (*SD*)] was lower than ICC gain LF, respectively, 0.38 (0.057) and 0.59 (0.078), *p* < 0.001. Also for phase, the corresponding ICC values were lower for VLF than for LF, 0.17 (0.13) and 0.39 (0.11), respectively, *p* = 0.001. For ARI-like methods the mean (*SD*) ICC results were 0.30 (0.12) and for the correlation-like 0.24 (0.21).

## Discussion

With this multi-center, multi-method study, we aimed to provide an internationally representative and broader evaluation of the reproducibility of many DCA assessment methods. By comparing real physiological measurements with those where physiological variability was reduced by use of surrogate data, we have been able to assess the contribution of physiological non-stationary to the reproducibility of DCA parameters. For surrogate data, with realistic CBFv signals generated from measured BP data, we had demonstrated good to excellent reproducibility for most DCA methods. We now hypothesized that in real recordings of BP and CBF, non-stationarity in the BP–CBF relationship would reduce reproducibility for these DCA methods.

We asked researchers from various centers with expertise in DCA to apply their DCA method(s) to a common dataset with repeated physiological measurements of BP and CBFv. Participating centers, and respective analytical methods, are representative of the literature on DCA assessment (Panerai et al., 1998a,b, 2000, 2003; Zhang et al., 1998; Torrence and Webster, 1999; Simpson et al., 2001; Liu and Allen, 2002; Mitsis et al., 2002, 2004, 2009; Liu et al., 2003, 2005; Muller et al., 2003; Reinhard et al., 2003; Grinsted et al., 2004; Marmarelis, 2004; Gommer et al., 2010; Peng et al., 2010; van Beek et al., 2010; Heskamp et al., 2013; Marmarelis et al., 2013, 2014a,b; Meel-van den Abeelen et al., 2014; Muller and Osterreich, 2014; Panerai, 2014; Caicedo et al., 2016).

### Main Findings

Two main outstanding findings came out of the study: (i) the reproducibility of most DCA metrics, independently of the analytical approach adopted, should be regarded as “poor,” given the prevailing values of ICC < 0.4 (Cicchetti, 1994) and (ii) physiological variability is likely to be the main reason for the degradation in reproducibility, when compared to results obtained from surrogate data (Sanders et al., 2018).

Strictly speaking, these results indicate that, at this moment, most DCA metrics do not meet criteria for individual and clinical use for diagnostic and/or monitoring purposes. Despite the high variability across DCA parameters, only TFA and ARX scored ICC results that could be categorized as “good” (ICC > 0.6, Figure 3) for approximately half of the gain metrics in the LF band (Cicchetti, 1994). As discussed in more detail below though, these findings need to be placed into perspective, taking into account methodological issues and current knowledge of the wider application of DCA assessment metrics.

### Methodological Considerations

Although indicative of the deterioration of DCA metrics, from what was obtained with surrogate data, to the case of “real” physiological measurements, the ICC can be misleading when estimated using only healthy subjects. Differently from the intra-subject standard error, the ICC takes into account both intra- and inter-subject variability. Given that healthy subjects would be expected to cluster around values indicative of a good working DCA, this would reduce inter-subject variability, in comparison with intra-subject variance, thus putting a bias toward reduced values of ICC. However, as can be observed in Figure 1, there was wide inter-subject variability, indicating that this alone cannot explain the low ICC results. Nonetheless, despite the indication that most DCA metrics have limited reproducibility, it would be premature to use our findings to put a halt on their use in physiological and clinical studies, before further research is conducted, ideally assessing the ICC for much larger cohorts of both patients and healthy individuals.

The analysis of physiological data presents large within and between subject variability, similar to what has been reported before in patient data (Gommer et al., 2010; van Beek et al., 2010; Elting et al., 2014; Smirl et al., 2015). Non-Gaussian distributions were corrected by the Box–Cox transformations (Box and Cox, 1964). The ICC values were much lower than what was found when these same methods were applied to analyze surrogate data (Sanders et al., 2018). In that study, physiological variability was reduced to only the BP signal, because the CBF signal was software-generated using the repeated BP signals as input. Even though realistic levels of noise were added to the generated CBF signal, all DCA methods demonstrated good to excellent reproducibility (ICC 0.6–1.00) on those surrogate data, whereas the majority of these same methods had poor reproducibility (ICC < 0.4) for the current dataset where both BP and CBF signals represented physiological data. One interpretation of these results is that the poor reproducibility of DCA is not solely explained because the methods provide poor accuracy or poor precision. With surrogate data, all methods showed accuracy and precision, leading to good reproducibility.

Comparable with results of Smirl et al. (2015), the highest ICC results were obtained with gain LF parameters, although Figure 2 shows that reproducibility differs for different gain values, with highest reproducibility for lower gain values. This is a proportional increase in variability, recognizable by the arrowhead shape in Figure 2. ICC for gain and phase parameters is decreased in VLF compared to LF, and may be explained by the lower coherence between BP and CBFv in VLF oscillations, resulting in wider confidence limits for VLF and lower ICC values. Comparing gain ICC results with phase, one can see decreased reproducibility in the phase results over both frequency bands. This does not immediately favor gain parameters as more suitable DCA metrics, since a lower ICC value for phase can be expected purely based on the definition and dependence between the two parameters (Bendat and Piersol, 1986). This explains that confidence limits will automatically be wider for phase compared to gain. We recommend to routinely plot confidence limits when creating TFA results.

To improve reproducibility, it may be beneficial to use measurement conditions where the DCA regulatory system is maximally activated, for example in sit-to-stand measurements (Simpson and Claassen, 2018a,b) or squat-stand measurements (Smirl et al., 2015). This may result in minimal gain values in the LF band and improve reproducibility. However, it remains an ongoing debate whether TFA gain is the most suitable parameter to reflect state of DCA, or if phase may be more physiologically relevant.

### Clinical Implications

Given the limited reproducibility shown by most indices of DCA, to what extent should we trust their use in clinical studies? This is a crucial question given the stage of research on DCA, with many centers advocating the use of DCA metrics in clinical decision-making and patient management. In this context, the results of this study might be a watershed. Until recently, the prevailing view has been that, among a plethora of DCA metrics, there could be one that could become a “gold standard” based on its reproducibility, as well as its sensitivity and specificity, to detect changes in DCA, either due to disease or physiological status. What this study is showing though, is that none of the methods in use could fulfill this role, at least not as reproducibility is concerned. Furthermore, the comparison between physiological and surrogate data also suggests that it is unlikely that other current or future methods will have an outstanding reproducibility either. The reason for this somber perspective lies with the growing awareness that regulation of CBF, not only in response to BP changes, but also due to changes in CO_2_ or neural stimulation, is a highly non-stationary phenomenon, thus requiring an entirely different conceptual paradigm to ascertain their clinical usefulness (Panerai, 2014). On the other hand, it is not all gloom and doom. Looking back into a vast literature, too extensive to be enumerated here, reporting on clinical applications of most of the DCA metrics included in this study, there is plenty of evidence to suggest their sensitivity to detect worsening DCA in a range of cerebrovascular and, increasingly, also systemic conditions. To study reproducibility in the presence of disease is a major challenge though, as patient conditions are either worsening or improving on a daily basis. Nevertheless, several follow up studies have been able to use diverse indices of DCA to describe the natural history of conditions like severe head injury (Czosnyka et al., 1997), ischemic stroke (Salinet et al., 2014), or intracerebral hemorrhage (Ma et al., 2016) which is also reassuring. Certainly much more research is needed, mainly to understand the nature of DCA non-stationarity and how this is affected by, and manifested in, clinical conditions, to improve the reliability and usefulness of DCA assessment for patient care.

### Limitations and Future Directions

Only methods that could be applied to short data segments (5 min) were evaluated; therefore, the correlation-like methods were underrepresented. The correlation-like methods clearly showed reduced reproducibility compared to the other categories (Figure 3) under these conditions.

It is difficult to select a suitable method to assess reproducibility of DCA analysis parameters. We selected ICC, although this method being sensitive to outliers. This has probably affected phase VLF results the strongest in a negative way, since high variability and outliers were most present in phase VLF.

The time interval differences between repeated measurements were not considered in the analysis. A dataset consisting of rest measurements was used, with limited BP fluctuations, resulting in a low power of BP and CBFv oscillations. At rest, cerebral perfusion is usually well maintained and DCA may not be activated, while during a physical challenge, when sufficient DCA functioning is crucial, will give more meaningful results (Simpson and Claassen, 2018a,b; Tzeng and Panerai, 2018a,b). Moreover, it will be relevant to add clinical data to the healthy controls to have a greater spread of inter-subject variability.

It could not yet be answered what the precise reason is for low reproducibility of DCA assessment in physiological data. It is necessary to study physiological variation in DCA function within individuals in repeated measurements. From a theoretical perspective, the variability in DCA results can be reduced in two ways: Increase the coherence or increase the number of averages (Bendat and Piersol, 1986; Halliday et al., 1995). To increase the coherence, oscillations could be induced and included in the measurement protocol. Increased coherence could also be achieved by selection of the data used for DCA analysis based on the power of BP oscillations. This line of investigation will be pursued as part of this wider project. To increase the number of averages, more or longer measurement protocols should be used, although duration of recordings is usually limited in most clinical settings.

Selecting the most promising DCA parameter is complex, since the most reproducible parameter is not necessarily the best parameter to reflect DCA status. Although there was not a single method that outperformed others both linear and non-linear, there are inter-method differences that are worth investigating. In particular, future studies could look to the influence of measurement length or increased oscillations in the measurement protocol or data selection (Simpson and Claassen, 2018a,b).

Furthermore, the question to answer is to what extent does reproducibility depend on autoregulation status. Are DCA parameters less reproducible in case of worse DCA status and functioning? One interesting and relatively easy next step could be to perform repeated measurements in hypercapnic data (Katsogridakis et al., 2013), as a model for impaired DCA, and compare these with repeated measurements in normocapnia to assess differences in reproducibility.

## Conclusion

The physiological nature of these measurements strongly reduced reproducibility of DCA when assessed in short data recordings in healthy subjects. This conclusion is not affected by the choice of analytical method used to derive different DCA metrics, or by local procedures in multiple international centers which participated in this study. Further investigation is needed to improve our understanding of how physiological variability affects DCA reproducibility in health and disease.

## Data Availability

The datasets generated for this study are available on request to the corresponding author.

## Ethics Statement

All subjects gave written informed consent in accordance with the Declaration of Helsinki. The six data providing centers and the ethical approval details: (1) JC: Radboudumc, Netherlands (ethical approval was given by the Local Medical Ethics Committee Arnhem–Nijmegen, Netherlands). (2) JE: University Medical Center Groningen, Netherlands (ethical approval was given by the Local Ethics Committee of the University Medical Centre Groningen). (3) EG: University Hospital Maastricht, Netherlands (ethical approval was given by the Medical Ethical Review Board of the Maastricht University Hospital/Maastricht University with METC reference no. 07-2-003). (4) RBP: Glenfield Hospital, Leicester, United Kingdom and University of Southampton, United Kingdom [ethical approval was given by the Research Ethics Committee of the National Research Ethics Service Southampton and Southwest Hampshire (10/H0502/1)]. (5) DMS: University of Southampton, United Kingdom [ethical approval was given by the National Research Ethics Service and Southampton University Hospitals NHS Trust (RHM HOS0199)]. (6) RZ: the Institute for Exercise and Environmental Medicine (IEEM), University of Texas Southwestern Medical Center, United States (ethical approval was given by the Institutional Review Boards of University of Texas Southwestern Medical Center and Texas Health Presbyterian Hospital of Dallas, TX, United States).

## Author Contributions

MS, JE, RBP, and JC developed the idea for the study and drafted the manuscript. All authors performed the data analyses, participated in revising the manuscript, and approved the final version of this paper prior to submission.

## Conflict of Interest Statement

The authors declare that the research was conducted in the absence of any commercial or financial relationships that could be construed as a potential conflict of interest.
