# Supplementary material for: Dynamic Cerebral Autoregulation Reproducibility Is Affected by Physiological Variability
Source: Front Physiol. 2019 Jul 9;10:865. doi: 10.3389/fphys.2019.00865 (PMC6634255; doi:10.3389/fphys.2019.00865)
Supplement: Supplementary file 1 [file Data_Sheet_1.docx]

| **Name** | **Institution** | **Country** | **Role** | **Centre**  **Number** |
| --- | --- | --- | --- | --- |
| E Borg-Seng-Shu  RC Nogueira | Department of Neurology, Hospital das Clinicas  University of Sao Paulo | Brazil | Analysis | 1 |
| VZ Marmarelis  DC Shin | Department of Biomedical Engineering  University of Southern California, Los Angeles | USA | Analysis | 2 |
| R Zhang  T Tarumi | IEEM, Presbyterian Hospital Dallas  University of Texas Southwestern Medical Center | USA | Analysis  Data Provider | 3 |
| RB Panerai | Department of Cardiovascular Sciences  University of Leicester | UK | Analysis  Data Provider  Trial Coordination | 4 |
| S van Huffel  A Caicedo | Department of Electronic Engineering (ESAT), STADIUS Center for Dynamical Systems, Signal Processing and Data Analytics, KU Leuven, Belgium; imec | Belgium | Analysis | 5 |
| M Müller | Department of Neurology  Luzerner Kantonsspital | Switzerland | Analysis | 6 |
| ED Gommer | Department of Clinical Neurophysiology  University Hospital Maastricht | Netherlands | Analysis  Data Provider | 7 |
| SJ Payne  A Mahdi | Department of Engineering Science  University of Oxford | UK | Analysis | 8 |
| JAHR Claassen  ML Sanders | Department of Geriatric Medicine  Radboud University Nijmegen | Netherlands | Analysis  Data Provider  Trial Coordination | 9 |
| DM Simpson  D Nikolic | Institute of Sound and Vibration Research  University of Southampton | UK | Analysis  Data Provider | 11 |
| JWJ Elting  M Aries | Department of Neurology  University Medical Center Groningen | Netherlands | Analysis  Data Provider  Trial Coordination | 12 |
| C Puppo  B Yelicich | Departamento de Emergencia, Hospital de ClínicasUniversidad de la República, Montevideo | Uruguay | Analysis | 13 |
| GD Mitsis  K Kostoglou | Department of Bioengineering  Department of Electrical, Computer and Software Engineering  McGill University, Montreal | Canada | Analysis | 14 |

**Table S1.** Participating centres and their roles

Centre 10 withdrew their results from analysis because their method was superseded by recent developments that would disadvantage their original approach.

**Figure S1.** Phase VLF results of TFA-like methods for repeated measurements. Top row: physiological data, bottom row: surrogate data. For each method group (TFA, Laguerre, Wavelet, ARX) the results of similar methods are combined (Table 1). TFA: black dots are 10 methods (rad), grey dots are 1 method (rad); Laguerre: 4 methods (cm/s/mmHg); Wavelet: 2 methods (cm/s/mmHg); ARX: 2 methods (cm/s/mmHg).

**Figure S2.** Phase LF results of TFA-like methods for repeated measurements. Top row: physiological data, bottom row: surrogate data. For each method group (TFA, Laguerre, Wavelet, IR-filter, ARX) the results of the similar method group are combined (Table 1). TFA: black dots are 10 methods (rad), grey dots are 1 method (rad); Laguerre: 4 methods (cm/s/mmHg); Wavelet: 2 methods (cm/s/mmHg); IR-filter: 2 methods (%/%); ARX: 2 methods (cm/s/mmHg).

**Figure S3.** Gain VLF results of TFA-like methods for repeated measurements. Top row: physiological data, bottom row: surrogate data. For each method group ((TFA, Laguerre, Wavelet, ARX) the results of similar methods are combined (Table 1). TFA: black dots are 10 methods (cm/s/mmHg), grey dots are 3 methods (%/% or %/mmHg); Laguerre: 4 methods (cm/s/mmHg); Wavelet: 1 method (cm/s/mmHg); ARX: 2 methods (cm/s/mmHg).

**Table S2 (a).** Gain VLF results per method.

|  |  | nr of cases | | | L | | | | | | R | | | | | | S | | | | | |
| --- | --- | --- | --- | --- | --- | --- | --- | --- | --- | --- | --- | --- | --- | --- | --- | --- | --- | --- | --- | --- | --- | --- |
| Gain VLF | Method | L | R | S | T1 | | | T2 | | | T1 | | | T2 | | | T1 | | | T2 | | |
| **TFA** | 1,1 | 54 | 68 | 22 | 0.69 | ± | 0.37 | 0.58 | ± | 0.20 | 0.68 | ± | 0.40 | 0.63 | ± | 0.24 | 0.22 | ± | 0.10 | 0.24 | ± | 0.11 |
| **Laguerre** | 2,1 | 55 | 71 | 22 | 0.52 | ± | 0.29 | 0.41 | ± | 0.15 | 0.49 | ± | 0.29 | 0.46 | ± | 0.24 | 0.17 | ± | 0.08 | 0.18 | ± | 0.09 |
| **Laguerre** | 2,2 | 55 | 71 | 22 | 0.49 | ± | 0.24 | 0.39 | ± | 0.14 | 0.46 | ± | 0.25 | 0.42 | ± | 0.16 | 0.17 | ± | 0.08 | 0.18 | ± | 0.09 |
| **TFA** | 3,1 | 55 | 71 | 22 | 0.64 | ± | 0.32 | 0.57 | ± | 0.23 | 0.64 | ± | 0.33 | 0.63 | ± | 0.26 | 0.26 | ± | 0.16 | 0.28 | ± | 0.14 |
| **TFA** | 3,2 | 55 | 71 | 22 | 1.11 | ± | 0.52 | 0.99 | ± | 0.34 | 1.14 | ± | 0.48 | 1.14 | ± | 0.50 | 0.53 | ± | 0.30 | 0.56 | ± | 0.26 |
| **TFA** | 5,1 | 55 | 71 | 22 | 0.63 | ± | 0.37 | 0.55 | ± | 0.22 | 0.62 | ± | 0.35 | 0.61 | ± | 0.27 | 0.23 | ± | 0.13 | 0.25 | ± | 0.12 |
| **TFA** | 6,1 | 55 | 71 | 22 | 0.36 | ± | 0.19 | 0.31 | ± | 0.18 | 0.34 | ± | 0.18 | 0.36 | ± | 0.23 | 0.23 | ± | 0.13 | 0.24 | ± | 0.13 |
| **TFA** | 7,1 | 55 | 71 | 22 | 0.56 | ± | 0.31 | 0.45 | ± | 0.18 | 0.55 | ± | 0.33 | 0.51 | ± | 0.25 | 0.18 | ± | 0.10 | 0.21 | ± | 0.11 |
| **TFA** | 9,1 | 55 | 71 | 22 | 0.60 | ± | 0.26 | 0.55 | ± | 0.19 | 0.60 | ± | 0.30 | 0.59 | ± | 0.22 | 0.28 | ± | 0.12 | 0.30 | ± | 0.11 |
| **TFA** | 11,1 | 55 | 71 | 22 | 0.55 | ± | 0.33 | 0.46 | ± | 0.21 | 0.54 | ± | 0.32 | 0.54 | ± | 0.29 | 0.19 | ± | 0.12 | 0.22 | ± | 0.11 |
| **TFA** | 11,2 | 55 | 71 | 22 | 0.97 | ± | 0.55 | 0.81 | ± | 0.33 | 0.97 | ± | 0.48 | 0.98 | ± | 0.56 | 0.39 | ± | 0.23 | 0.44 | ± | 0.20 |
| **TFA** | 11,3 | 55 | 71 | 22 | 0.84 | ± | 0.45 | 0.69 | ± | 0.28 | 0.85 | ± | 0.40 | 0.85 | ± | 0.47 | 0.35 | ± | 0.18 | 0.38 | ± | 0.18 |
| **TFA** | 12,1 | 46 | 58 | 14 | 0.82 | ± | 0.53 | 0.73 | ± | 0.35 | 0.87 | ± | 0.78 | 0.77 | ± | 0.38 | 0.35 | ± | 0.16 | 0.36 | ± | 0.18 |
| **Wavelets** | 12,3 | 54 | 67 | 19 | 0.91 | ± | 0.46 | 0.79 | ± | 0.36 | 0.90 | ± | 0.50 | 0.92 | ± | 0.43 | 0.35 | ± | 0.18 | 0.42 | ± | 0.18 |
| **TFA** | 13,1 | 55 | 71 | 22 | 0.56 | ± | 0.21 | 0.53 | ± | 0.24 | 0.55 | ± | 0.23 | 0.57 | ± | 0.28 | 0.35 | ± | 0.17 | 0.41 | ± | 0.22 |
| **ARX** | 14,1 | 55 | 71 | 22 | 0.48 | ± | 0.27 | 0.42 | ± | 0.18 | 0.48 | ± | 0.31 | 0.46 | ± | 0.22 | 0.22 | ± | 0.10 | 0.23 | ± | 0.11 |
| **ARX** | 14,2 | 55 | 71 | 22 | 0.47 | ± | 0.30 | 0.41 | ± | 0.17 | 0.47 | ± | 0.32 | 0.44 | ± | 0.19 | 0.22 | ± | 0.11 | 0.22 | ± | 0.11 |
| **Laguerre** | 14,3 | 55 | 71 | 22 | 0.51 | ± | 0.29 | 0.45 | ± | 0.20 | 0.49 | ± | 0.27 | 0.50 | ± | 0.25 | 0.19 | ± | 0.10 | 0.21 | ± | 0.12 |
| **Laguerre** | 14,4 | 55 | 71 | 22 | 0.50 | ± | 0.33 | 0.44 | ± | 0.20 | 0.49 | ± | 0.28 | 0.48 | ± | 0.22 | 0.19 | ± | 0.11 | 0.23 | ± | 0.13 |
| **TFA** | 14,5 | 55 | 71 | 22 | 0.61 | ± | 0.37 | 0.55 | ± | 0.23 | 0.63 | ± | 0.37 | 0.62 | ± | 0.28 | 0.23 | ± | 0.14 | 0.28 | ± | 0.16 |
|  |  |  |  |  |  |  |  |  |  |  |  |  |  |  |  |  |  |  |  |  |  |  |

Method as listed in Table 1. Nr of cases: for left (L), right(R) and surrogate data (S). Data for repeated measurements (T1 and T2, mean ± SD)

**Table S2(b).** Gain LF results per method.

|  |  |  |  |  |  |  |  |  |  |  |  |  |  |  |  |  |  |  |  |  |  |  |
| --- | --- | --- | --- | --- | --- | --- | --- | --- | --- | --- | --- | --- | --- | --- | --- | --- | --- | --- | --- | --- | --- | --- |
|  |  | nr of cases | | | L | | | | | | R | | | | | | S | | | | | |
| Gain LF | Method | L | R | S | T1 | | | T2 | | | T1 | | | T2 | | | T1 | | | T2 | | |
| **TFA** | 1,1 | 54 | 68 | 22 | 0.99 | ± | 0.46 | 0.90 | ± | 0.36 | 0.98 | ± | 0.52 | 0.91 | ± | 0.35 | 0.27 | ± | 0.07 | 0.29 | ± | 0.09 |
| **Laguerre** | 2,1 | 55 | 71 | 22 | 0.85 | ± | 0.35 | 0.77 | ± | 0.28 | 0.82 | ± | 0.39 | 0.80 | ± | 0.36 | 0.26 | ± | 0.09 | 0.28 | ± | 0.12 |
| **Laguerre** | 2,2 | 55 | 71 | 22 | 0.84 | ± | 0.37 | 0.75 | ± | 0.28 | 0.81 | ± | 0.40 | 0.77 | ± | 0.28 | 0.26 | ± | 0.10 | 0.28 | ± | 0.12 |
| **TFA** | 3,1 | 55 | 71 | 22 | 0.95 | ± | 0.50 | 0.87 | ± | 0.36 | 0.93 | ± | 0.55 | 0.86 | ± | 0.33 | 0.26 | ± | 0.08 | 0.28 | ± | 0.10 |
| **TFA** | 3,2 | 55 | 71 | 22 | 1.64 | ± | 0.79 | 1.52 | ± | 0.51 | 1.61 | ± | 0.73 | 1.53 | ± | 0.46 | 0.53 | ± | 0.13 | 0.56 | ± | 0.14 |
| **TFA** | 5,1 | 55 | 71 | 22 | 0.90 | ± | 0.44 | 0.83 | ± | 0.37 | 0.88 | ± | 0.54 | 0.80 | ± | 0.34 | 0.26 | ± | 0.09 | 0.28 | ± | 0.11 |
| **TFA** | 6,1 | 55 | 71 | 22 | 0.63 | ± | 0.31 | 0.57 | ± | 0.28 | 0.61 | ± | 0.36 | 0.54 | ± | 0.26 | 0.21 | ± | 0.13 | 0.22 | ± | 0.12 |
| **TFA** | 7,1 | 55 | 71 | 22 | 0.85 | ± | 0.46 | 0.77 | ± | 0.32 | 0.82 | ± | 0.52 | 0.75 | ± | 0.30 | 0.26 | ± | 0.09 | 0.28 | ± | 0.11 |
| **TFA** | 9,1 | 55 | 71 | 22 | 0.86 | ± | 0.43 | 0.78 | ± | 0.34 | 0.83 | ± | 0.52 | 0.76 | ± | 0.31 | 0.26 | ± | 0.09 | 0.28 | ± | 0.11 |
| **TFA** | 11,1 | 55 | 71 | 22 | 0.90 | ± | 0.45 | 0.83 | ± | 0.36 | 0.88 | ± | 0.52 | 0.81 | ± | 0.33 | 0.26 | ± | 0.09 | 0.28 | ± | 0.10 |
| **TFA** | 11,2 | 55 | 71 | 22 | 1.56 | ± | 0.71 | 1.45 | ± | 0.52 | 1.52 | ± | 0.69 | 1.44 | ± | 0.46 | 0.53 | ± | 0.14 | 0.56 | ± | 0.14 |
| **TFA** | 11,3 | 55 | 71 | 22 | 1.34 | ± | 0.53 | 1.22 | ± | 0.40 | 1.33 | ± | 0.49 | 1.24 | ± | 0.37 | 0.49 | ± | 0.10 | 0.49 | ± | 0.09 |
| **IR filter** | 11,4 | 55 | 71 | 22 | 1.49 | ± | 0.55 | 1.30 | ± | 0.42 | 1.44 | ± | 0.49 | 1.38 | ± | 0.51 | 0.50 | ± | 0.11 | 0.52 | ± | 0.13 |
| **IR filter** | 11,6 | 55 | 71 | 22 | 1.46 | ± | 0.55 | 1.26 | ± | 0.37 | 1.41 | ± | 0.50 | 1.35 | ± | 0.41 | 0.51 | ± | 0.12 | 0.54 | ± | 0.16 |
| **TFA** | 12,1 | 54 | 71 | 22 | 0.91 | ± | 0.47 | 0.83 | ± | 0.34 | 0.90 | ± | 0.56 | 0.83 | ± | 0.34 | 0.26 | ± | 0.09 | 0.28 | ± | 0.11 |
| **Wavelets** | 12,3 | 55 | 71 | 22 | 1.04 | ± | 0.50 | 0.96 | ± | 0.37 | 1.06 | ± | 0.59 | 0.98 | ± | 0.37 | 0.40 | ± | 0.18 | 0.45 | ± | 0.19 |
| **TFA** | 13,1 | 55 | 71 | 22 | 0.79 | ± | 0.34 | 0.73 | ± | 0.28 | 0.78 | ± | 0.40 | 0.70 | ± | 0.26 | 0.38 | ± | 0.11 | 0.39 | ± | 0.11 |
| **ARX** | 14,1 | 55 | 71 | 22 | 0.81 | ± | 0.39 | 0.74 | ± | 0.27 | 0.77 | ± | 0.45 | 0.72 | ± | 0.26 | 0.26 | ± | 0.09 | 0.28 | ± | 0.11 |
| **ARX** | 14,2 | 55 | 71 | 22 | 0.81 | ± | 0.39 | 0.73 | ± | 0.26 | 0.77 | ± | 0.45 | 0.73 | ± | 0.26 | 0.26 | ± | 0.09 | 0.28 | ± | 0.11 |
| **Laguerre** | 14,3 | 55 | 71 | 22 | 0.88 | ± | 0.52 | 0.77 | ± | 0.33 | 0.85 | ± | 0.56 | 0.79 | ± | 0.32 | 0.27 | ± | 0.11 | 0.28 | ± | 0.14 |
| **Laguerre** | 14,4 | 55 | 71 | 22 | 0.89 | ± | 0.49 | 0.75 | ± | 0.33 | 0.83 | ± | 0.52 | 0.74 | ± | 0.33 | 0.27 | ± | 0.11 | 0.29 | ± | 0.13 |
| **TFA** | 14,5 | 55 | 71 | 22 | 0.88 | ± | 0.44 | 0.81 | ± | 0.35 | 0.87 | ± | 0.55 | 0.79 | ± | 0.33 | 0.26 | ± | 0.10 | 0.28 | ± | 0.11 |
|  |  |  |  |  |  |  |  |  |  |  |  |  |  |  |  |  |  |  |  |  |  |  |

Method as listed in Table 1. Nr of cases: for left (L), right(R) and surrogate data (S). Data for repeated measurements (T1 and T2, mean ± SD)

**Table S2(c).** Gain HF results per method.

|  |  |  |  |  |  |  |  |  |  |  |  |  |  |  |  |  |  |  |  |  |  |  |
| --- | --- | --- | --- | --- | --- | --- | --- | --- | --- | --- | --- | --- | --- | --- | --- | --- | --- | --- | --- | --- | --- | --- |
|  |  | nr of cases | | | L | | | | | | R | | | | | | S | | | | | |
| Gain HF | Method | L | R | S | T1 | | | T2 | | | T1 | | | T2 | | | T1 | | | T2 | | |
| **TFA** | 1,1 | 54 | 68 | 22 | 1.01 | ± | 0.41 | 0.91 | ± | 0.32 | 0.95 | ± | 0.43 | 0.94 | ± | 0.37 | 0.29 | ± | 0.06 | 0.31 | ± | 0.08 |
| **Laguerre** | 2,1 | 55 | 71 | 22 | 1.24 | ± | 0.57 | 1.07 | ± | 0.41 | 1.20 | ± | 0.66 | 1.16 | ± | 0.52 | 0.33 | ± | 0.08 | 0.37 | ± | 0.13 |
| **Laguerre** | 2,2 | 55 | 71 | 22 | 1.22 | ± | 0.54 | 1.06 | ± | 0.40 | 1.17 | ± | 0.64 | 1.12 | ± | 0.44 | 0.34 | ± | 0.09 | 0.37 | ± | 0.13 |
| **TFA** | 3,1 | 55 | 71 | 22 | 1.06 | ± | 0.48 | 0.95 | ± | 0.35 | 0.99 | ± | 0.49 | 0.98 | ± | 0.40 | 0.29 | ± | 0.06 | 0.31 | ± | 0.08 |
| **TFA** | 3,2 | 55 | 71 | 22 | 1.85 | ± | 0.79 | 1.69 | ± | 0.55 | 1.73 | ± | 0.68 | 1.76 | ± | 0.57 | 0.58 | ± | 0.10 | 0.62 | ± | 0.11 |
| **TFA** | 5,1 | 55 | 71 | 22 | 1.04 | ± | 0.45 | 0.93 | ± | 0.35 | 0.96 | ± | 0.46 | 0.96 | ± | 0.37 | 0.28 | ± | 0.06 | 0.31 | ± | 0.08 |
| **TFA** | 6,1 | 55 | 71 | 22 | 0.92 | ± | 0.41 | 0.81 | ± | 0.31 | 0.83 | ± | 0.43 | 0.83 | ± | 0.35 | 0.26 | ± | 0.06 | 0.28 | ± | 0.09 |
| **TFA** | 7,1 | 55 | 71 | 22 | 0.99 | ± | 0.44 | 0.88 | ± | 0.33 | 0.91 | ± | 0.48 | 0.90 | ± | 0.34 | 0.28 | ± | 0.06 | 0.31 | ± | 0.08 |
| **TFA** | 9,1 | 55 | 71 | 22 | 1.02 | ± | 0.44 | 0.90 | ± | 0.34 | 0.94 | ± | 0.46 | 0.93 | ± | 0.36 | 0.28 | ± | 0.06 | 0.31 | ± | 0.08 |
| **TFA** | 11,1 | 55 | 71 | 22 | 0.96 | ± | 0.39 | 0.86 | ± | 0.32 | 0.89 | ± | 0.43 | 0.89 | ± | 0.35 | 0.28 | ± | 0.06 | 0.31 | ± | 0.08 |
| **TFA** | 11,2 | 55 | 71 | 22 | 1.69 | ± | 0.65 | 1.53 | ± | 0.52 | 1.57 | ± | 0.58 | 1.60 | ± | 0.52 | 0.58 | ± | 0.09 | 0.61 | ± | 0.10 |
| **TFA** | 11,3 | 55 | 71 | 22 | 1.47 | ± | 0.56 | 1.29 | ± | 0.44 | 1.39 | ± | 0.49 | 1.39 | ± | 0.45 | 0.53 | ± | 0.05 | 0.54 | ± | 0.05 |
| **TFA** | 12,1 | 55 | 71 | 22 | 1.13 | ± | 0.48 | 1.02 | ± | 0.40 | 1.08 | ± | 0.53 | 1.05 | ± | 0.42 | 0.28 | ± | 0.06 | 0.31 | ± | 0.09 |
| **Wavelets** | 12,3 | 55 | 71 | 22 | 1.18 | ± | 0.50 | 1.15 | ± | 0.45 | 1.19 | ± | 0.58 | 1.15 | ± | 0.45 | 0.31 | ± | 0.08 | 0.37 | ± | 0.20 |
| **TFA** | 13,1 | 55 | 71 | 22 | 0.84 | ± | 0.32 | 0.78 | ± | 0.23 | 0.79 | ± | 0.32 | 0.77 | ± | 0.24 | 0.43 | ± | 0.09 | 0.46 | ± | 0.13 |
| **ARX** | 14,1 | 55 | 71 | 22 | 0.97 | ± | 0.46 | 0.88 | ± | 0.32 | 0.92 | ± | 0.49 | 0.88 | ± | 0.33 | 0.28 | ± | 0.06 | 0.30 | ± | 0.08 |
| **ARX** | 14,2 | 55 | 71 | 22 | 0.97 | ± | 0.45 | 0.88 | ± | 0.31 | 0.92 | ± | 0.50 | 0.89 | ± | 0.33 | 0.28 | ± | 0.06 | 0.30 | ± | 0.08 |
| **Laguerre** | 14,3 | 55 | 71 | 22 | 1.02 | ± | 0.64 | 0.93 | ± | 0.46 | 0.99 | ± | 0.66 | 0.98 | ± | 0.46 | 0.27 | ± | 0.08 | 0.29 | ± | 0.12 |
| **Laguerre** | 14,4 | 55 | 71 | 22 | 1.06 | ± | 0.65 | 0.90 | ± | 0.46 | 0.98 | ± | 0.62 | 0.92 | ± | 0.47 | 0.28 | ± | 0.10 | 0.31 | ± | 0.10 |
| **TFA** | 14,5 | 55 | 71 | 22 | 1.04 | ± | 0.45 | 0.93 | ± | 0.34 | 0.96 | ± | 0.47 | 0.96 | ± | 0.37 | 0.28 | ± | 0.06 | 0.31 | ± | 0.08 |
|  |  |  |  |  |  |  |  |  |  |  |  |  |  |  |  |  |  |  |  |  |  |  |

Method as listed in Table 1. Nr of cases: for left (L), right(R) and surrogate data (S). Data for repeated measurements (T1 and T2, mean ± SD)

**Table S2(d).** Phase VLF results per method.

|  |  |  |  |  |  |  |  |  |  |  |  |  |  |  |  |  |  |  |  |  |  |  |
| --- | --- | --- | --- | --- | --- | --- | --- | --- | --- | --- | --- | --- | --- | --- | --- | --- | --- | --- | --- | --- | --- | --- |
|  |  | nr of cases | | | L | | | | | | R | | | | | | S | | | | | |
| Phase VLF | Method | L | R | S | T1 | | | T2 | | | T1 | | | T2 | | | T1 | | | T2 | | |
| **TFA** | 1,1 | 54 | 68 | 22 | 0.71 | ± | 0.35 | 0.69 | ± | 0.32 | 0.71 | ± | 0.36 | 0.64 | ± | 0.35 | 0.37 | ± | 0.37 | 0.46 | ± | 0.43 |
| **Laguerre** | 2,1 | 55 | 71 | 22 | 0.79 | ± | 0.55 | 0.95 | ± | 0.48 | 0.84 | ± | 0.57 | 0.87 | ± | 0.54 | 0.74 | ± | 0.63 | 0.67 | ± | 0.54 |
| **Laguerre** | 2,2 | 55 | 71 | 22 | 0.85 | ± | 0.52 | 0.94 | ± | 0.46 | 0.88 | ± | 0.53 | 0.92 | ± | 0.51 | 0.72 | ± | 0.63 | 0.70 | ± | 0.58 |
| **TFA** | 3,1 | 55 | 71 | 22 | 0.77 | ± | 0.40 | 0.79 | ± | 0.52 | 0.84 | ± | 0.45 | 0.82 | ± | 0.47 | 0.52 | ± | 0.64 | 0.49 | ± | 0.49 |
| **TFA** | 5,1 | 55 | 71 | 22 | 0.86 | ± | 0.47 | 0.81 | ± | 0.57 | 0.79 | ± | 0.52 | 0.79 | ± | 0.53 | 0.51 | ± | 0.74 | 0.51 | ± | 0.56 |
| **TFA** | 6,1 | 55 | 71 | 22 | 0.86 | ± | 0.32 | 0.88 | ± | 0.28 | 0.88 | ± | 0.30 | 0.89 | ± | 0.29 | 0.63 | ± | 0.31 | 0.62 | ± | 0.30 |
| **TFA** | 7,1 | 55 | 71 | 22 | 0.86 | ± | 0.53 | 0.86 | ± | 0.57 | 0.75 | ± | 0.68 | 0.87 | ± | 0.73 | 0.71 | ± | 0.67 | 0.60 | ± | 0.59 |
| **Wavelets** | 8,2 | 55 | 71 | 22 | 0.97 | ± | 0.49 | 1.03 | ± | 0.59 | 0.87 | ± | 0.66 | 1.06 | ± | 0.65 | 0.69 | ± | 0.86 | 0.57 | ± | 0.57 |
| **TFA** | 9,1 | 55 | 71 | 21 | 0.93 | ± | 0.36 | 0.94 | ± | 0.35 | 0.94 | ± | 0.34 | 0.94 | ± | 0.37 | 0.76 | ± | 0.61 | 0.73 | ± | 0.61 |
| **TFA** | 11,1 | 55 | 69 | 21 | 1.01 | ± | 0.42 | 1.09 | ± | 0.46 | 1.06 | ± | 0.44 | 1.06 | ± | 0.48 | 0.86 | ± | 0.63 | 0.83 | ± | 0.57 |
| **TFA** | 11,2 | 55 | 69 | 21 | 1.01 | ± | 0.42 | 1.09 | ± | 0.46 | 1.06 | ± | 0.44 | 1.06 | ± | 0.48 | 0.86 | ± | 0.63 | 0.83 | ± | 0.57 |
| **TFA** | 12,1 | 46 | 58 | 14 | 0.92 | ± | 0.46 | 1.00 | ± | 0.45 | 0.90 | ± | 0.52 | 0.97 | ± | 0.46 | 0.70 | ± | 0.61 | 0.47 | ± | 0.60 |
| **Wavelets** | 12,3 | 54 | 67 | 19 | 0.80 | ± | 0.72 | 1.06 | ± | 0.50 | 0.93 | ± | 0.53 | 1.00 | ± | 0.63 | 0.71 | ± | 0.56 | 0.62 | ± | 0.80 |
| **TFA** | 13,1 | 55 | 71 | 22 | 0.62 | ± | 0.49 | 0.50 | ± | 0.50 | 0.59 | ± | 0.51 | 0.59 | ± | 0.49 | 0.14 | ± | 0.48 | 0.29 | ± | 0.58 |
| **ARX** | 14,1 | 55 | 71 | 22 | 1.03 | ± | 0.47 | 1.07 | ± | 0.45 | 0.98 | ± | 0.52 | 0.97 | ± | 0.54 | 0.80 | ± | 0.56 | 0.75 | ± | 0.58 |
| **ARX** | 14,2 | 55 | 71 | 22 | 1.05 | ± | 0.46 | 1.05 | ± | 0.39 | 1.01 | ± | 0.49 | 1.04 | ± | 0.42 | 0.77 | ± | 0.54 | 0.73 | ± | 0.65 |
| **Laguerre** | 14,3 | 55 | 71 | 22 | 0.79 | ± | 0.49 | 0.82 | ± | 0.54 | 0.70 | ± | 0.61 | 0.79 | ± | 0.53 | 0.73 | ± | 0.58 | 0.56 | ± | 0.61 |
| **Laguerre** | 14,4 | 55 | 71 | 22 | 0.76 | ± | 0.48 | 0.83 | ± | 0.53 | 0.77 | ± | 0.49 | 0.89 | ± | 0.56 | 0.73 | ± | 0.59 | 0.58 | ± | 0.56 |
| **TFA** | 14,5 | 55 | 71 | 22 | 0.85 | ± | 0.44 | 0.80 | ± | 0.58 | 0.81 | ± | 0.51 | 0.82 | ± | 0.53 | 0.52 | ± | 0.67 | 0.45 | ± | 0.57 |
|  |  |  |  |  |  |  |  |  |  |  |  |  |  |  |  |  |  |  |  |  |  |  |

Method as listed in Table 1. Nr of cases: for left (L), right(R) and surrogate data (S). Data for repeated measurements (T1 and T2, mean ± SD)

**Table S2(e).** Phase LF results per method.

|  |  |  |  |  |  |  |  |  |  |  |  |  |  |  |  |  |  |  |  |  |  |  |
| --- | --- | --- | --- | --- | --- | --- | --- | --- | --- | --- | --- | --- | --- | --- | --- | --- | --- | --- | --- | --- | --- | --- |
|  |  | nr of cases | | | L | | | | | | R | | | | | | S | | | | | |
| Phase LF | Method | L | R | S | T1 | | | T2 | | | T1 | | | T2 | | | T1 | | | T2 | | |
| **TFA** | 1,1 | 54 | 68 | 22 | 0.49 | ± | 0.26 | 0.50 | ± | 0.21 | 0.48 | ± | 0.25 | 0.47 | ± | 0.24 | 0.33 | ± | 0.43 | 0.34 | ± | 0.42 |
| **Laguerre** | 2,1 | 55 | 71 | 22 | 0.64 | ± | 0.25 | 0.69 | ± | 0.24 | 0.66 | ± | 0.28 | 0.67 | ± | 0.28 | 0.54 | ± | 0.49 | 0.51 | ± | 0.45 |
| **Laguerre** | 2,2 | 55 | 71 | 22 | 0.67 | ± | 0.24 | 0.70 | ± | 0.22 | 0.68 | ± | 0.26 | 0.69 | ± | 0.26 | 0.53 | ± | 0.48 | 0.52 | ± | 0.47 |
| **TFA** | 3,1 | 55 | 71 | 22 | 0.65 | ± | 0.22 | 0.66 | ± | 0.23 | 0.64 | ± | 0.25 | 0.63 | ± | 0.23 | 0.45 | ± | 0.50 | 0.44 | ± | 0.49 |
| **TFA** | 5,1 | 55 | 71 | 22 | 0.70 | ± | 0.24 | 0.71 | ± | 0.24 | 0.66 | ± | 0.32 | 0.72 | ± | 0.26 | 0.49 | ± | 0.58 | 0.50 | ± | 0.52 |
| **TFA** | 6,1 | 55 | 71 | 22 | 0.72 | ± | 0.24 | 0.75 | ± | 0.21 | 0.74 | ± | 0.27 | 0.76 | ± | 0.24 | 0.53 | ± | 0.53 | 0.52 | ± | 0.49 |
| **TFA** | 7,1 | 55 | 71 | 22 | 0.73 | ± | 0.23 | 0.74 | ± | 0.21 | 0.72 | ± | 0.25 | 0.74 | ± | 0.22 | 0.50 | ± | 0.54 | 0.50 | ± | 0.49 |
| **Wavelets** | 8,2 | 55 | 71 | 22 | 0.76 | ± | 0.24 | 0.79 | ± | 0.21 | 0.76 | ± | 0.24 | 0.78 | ± | 0.25 | 0.50 | ± | 0.54 | 0.51 | ± | 0.49 |
| **TFA** | 9,1 | 55 | 71 | 22 | 0.74 | ± | 0.23 | 0.76 | ± | 0.20 | 0.73 | ± | 0.25 | 0.76 | ± | 0.22 | 0.52 | ± | 0.55 | 0.53 | ± | 0.49 |
| **TFA** | 11,1 | 55 | 71 | 22 | 0.69 | ± | 0.21 | 0.70 | ± | 0.19 | 0.70 | ± | 0.23 | 0.69 | ± | 0.21 | 0.46 | ± | 0.52 | 0.47 | ± | 0.47 |
| **TFA** | 11,2 | 55 | 71 | 22 | 0.69 | ± | 0.21 | 0.70 | ± | 0.19 | 0.70 | ± | 0.23 | 0.69 | ± | 0.21 | 0.46 | ± | 0.52 | 0.47 | ± | 0.47 |
| **IR filter** | 11,4 | 55 | 71 | 21 | 0.58 | ± | 0.21 | 0.63 | ± | 0.18 | 0.60 | ± | 0.24 | 0.61 | ± | 0.22 | 0.47 | ± | 0.44 | 0.46 | ± | 0.41 |
| **IR filter** | 11,6 | 55 | 71 | 21 | 0.58 | ± | 0.21 | 0.62 | ± | 0.19 | 0.61 | ± | 0.23 | 0.61 | ± | 0.23 | 0.47 | ± | 0.43 | 0.48 | ± | 0.41 |
| **TFA** | 12,1 | 54 | 71 | 22 | 0.73 | ± | 0.22 | 0.77 | ± | 0.21 | 0.71 | ± | 0.27 | 0.75 | ± | 0.28 | 0.51 | ± | 0.56 | 0.52 | ± | 0.53 |
| **Wavelets** | 12,3 | 55 | 71 | 22 | 1.05 | ± | 0.32 | 1.11 | ± | 0.29 | 1.08 | ± | 0.29 | 1.15 | ± | 0.32 | 0.91 | ± | 0.57 | 0.82 | ± | 0.51 |
| **TFA** | 13,1 | 55 | 71 | 22 | 0.56 | ± | 0.25 | 0.54 | ± | 0.24 | 0.50 | ± | 0.27 | 0.51 | ± | 0.24 | 0.17 | ± | 0.23 | 0.18 | ± | 0.24 |
| **ARX** | 14,1 | 55 | 71 | 22 | 0.74 | ± | 0.33 | 0.74 | ± | 0.26 | 0.68 | ± | 0.30 | 0.70 | ± | 0.28 | 0.48 | ± | 0.54 | 0.51 | ± | 0.52 |
| **ARX** | 14,2 | 55 | 71 | 22 | 0.71 | ± | 0.27 | 0.72 | ± | 0.23 | 0.70 | ± | 0.31 | 0.71 | ± | 0.29 | 0.49 | ± | 0.54 | 0.51 | ± | 0.53 |
| **Laguerre** | 14,3 | 55 | 71 | 22 | 0.64 | ± | 0.36 | 0.60 | ± | 0.37 | 0.55 | ± | 0.47 | 0.63 | ± | 0.36 | 0.42 | ± | 0.56 | 0.37 | ± | 0.61 |
| **Laguerre** | 14,4 | 55 | 71 | 22 | 0.64 | ± | 0.33 | 0.59 | ± | 0.39 | 0.59 | ± | 0.37 | 0.65 | ± | 0.43 | 0.41 | ± | 0.60 | 0.43 | ± | 0.57 |
| **TFA** | 14,5 | 55 | 71 | 22 | 0.71 | ± | 0.27 | 0.73 | ± | 0.25 | 0.70 | ± | 0.28 | 0.74 | ± | 0.28 | 0.51 | ± | 0.58 | 0.50 | ± | 0.52 |
|  |  |  |  |  |  |  |  |  |  |  |  |  |  |  |  |  |  |  |  |  |  |  |

Method as listed in Table 1. Nr of cases: for left (L), right(R) and surrogate data (S). Data for repeated measurements (T1 and T2, mean ± SD)

**Table S2(f).** Phase HF results per method.

|  |  |  |  |  |  |  |  |  |  |  |  |  |  |  |  |  |  |  |  |  |  |  |
| --- | --- | --- | --- | --- | --- | --- | --- | --- | --- | --- | --- | --- | --- | --- | --- | --- | --- | --- | --- | --- | --- | --- |
|  |  | nr of cases | | | L | | | | | | R | | | | | | S | | | | | |
| Phase HF | Method | L | R | S | T1 | | | T2 | | | T1 | | | T2 | | | T1 | | | T2 | | |
| **TFA** | 1,1 | 54 | 68 | 22 | 0.03 | ± | 0.27 | 0.02 | ± | 0.25 | 0.02 | ± | 0.28 | 0.01 | ± | 0.27 | 0.12 | ± | 0.19 | 0.12 | ± | 0.19 |
| **Laguerre** | 2,1 | 55 | 71 | 22 | 0.06 | ± | 0.24 | 0.02 | ± | 0.25 | 0.04 | ± | 0.27 | 0.04 | ± | 0.29 | -0.09 | ± | 0.38 | -0.07 | ± | 0.38 |
| **Laguerre** | 2,2 | 55 | 71 | 22 | 0.06 | ± | 0.24 | 0.03 | ± | 0.25 | 0.04 | ± | 0.26 | 0.03 | ± | 0.29 | -0.07 | ± | 0.36 | -0.06 | ± | 0.36 |
| **TFA** | 3,1 | 55 | 71 | 22 | 0.13 | ± | 0.28 | 0.14 | ± | 0.26 | 0.11 | ± | 0.29 | 0.10 | ± | 0.28 | 0.18 | ± | 0.26 | 0.17 | ± | 0.26 |
| **TFA** | 5,1 | 55 | 71 | 22 | 0.16 | ± | 0.25 | 0.16 | ± | 0.22 | 0.14 | ± | 0.26 | 0.12 | ± | 0.21 | 0.17 | ± | 0.26 | 0.17 | ± | 0.26 |
| **TFA** | 6,1 | 55 | 71 | 22 | 0.41 | ± | 0.21 | 0.42 | ± | 0.19 | 0.46 | ± | 0.26 | 0.42 | ± | 0.21 | 0.18 | ± | 0.27 | 0.18 | ± | 0.26 |
| **TFA** | 7,1 | 55 | 71 | 22 | 0.19 | ± | 0.25 | 0.19 | ± | 0.21 | 0.19 | ± | 0.30 | 0.17 | ± | 0.26 | 0.18 | ± | 0.27 | 0.19 | ± | 0.27 |
| **Wavelets** | 8,2 | 55 | 71 | 22 | 0.29 | ± | 0.29 | 0.26 | ± | 0.24 | 0.27 | ± | 0.32 | 0.24 | ± | 0.26 | 0.21 | ± | 0.30 | 0.23 | ± | 0.31 |
| **TFA** | 9,1 | 54 | 71 | 22 | 0.40 | ± | 0.24 | 0.39 | ± | 0.16 | 0.41 | ± | 0.26 | 0.40 | ± | 0.19 | 0.18 | ± | 0.27 | 0.18 | ± | 0.27 |
| **TFA** | 11,1 | 54 | 70 | 22 | 0.32 | ± | 0.22 | 0.33 | ± | 0.21 | 0.37 | ± | 0.29 | 0.39 | ± | 0.26 | 0.12 | ± | 0.18 | 0.12 | ± | 0.18 |
| **TFA** | 11,2 | 54 | 70 | 22 | 0.32 | ± | 0.22 | 0.33 | ± | 0.21 | 0.37 | ± | 0.29 | 0.39 | ± | 0.26 | 0.12 | ± | 0.18 | 0.12 | ± | 0.18 |
| **TFA** | 12,1 | 55 | 71 | 22 | 0.33 | ± | 0.31 | 0.33 | ± | 0.27 | 0.34 | ± | 0.37 | 0.26 | ± | 0.27 | 0.24 | ± | 0.35 | 0.25 | ± | 0.35 |
| **Wavelets** | 12,3 | 55 | 71 | 22 | 0.78 | ± | 0.29 | 0.80 | ± | 0.27 | 0.77 | ± | 0.31 | 0.78 | ± | 0.27 | 0.48 | ± | 0.44 | 0.50 | ± | 0.43 |
| **TFA** | 13,1 | 55 | 71 | 22 | 0.11 | ± | 0.15 | 0.09 | ± | 0.13 | 0.08 | ± | 0.15 | 0.08 | ± | 0.15 | 0.06 | ± | 0.06 | 0.06 | ± | 0.10 |
| **ARX** | 14,1 | 55 | 71 | 22 | 0.15 | ± | 0.21 | 0.13 | ± | 0.17 | 0.11 | ± | 0.18 | 0.12 | ± | 0.19 | 0.17 | ± | 0.26 | 0.17 | ± | 0.26 |
| **ARX** | 14,2 | 55 | 71 | 22 | 0.15 | ± | 0.28 | 0.13 | ± | 0.17 | 0.13 | ± | 0.29 | 0.12 | ± | 0.19 | 0.17 | ± | 0.26 | 0.17 | ± | 0.26 |
| **Laguerre** | 14,3 | 55 | 71 | 22 | 0.20 | ± | 0.45 | 0.12 | ± | 0.26 | 0.13 | ± | 0.37 | 0.13 | ± | 0.25 | 0.03 | ± | 0.33 | 0.03 | ± | 0.35 |
| **Laguerre** | 14,4 | 55 | 71 | 22 | 0.16 | ± | 0.35 | 0.10 | ± | 0.28 | 0.11 | ± | 0.37 | 0.19 | ± | 0.46 | 0.08 | ± | 0.35 | 0.07 | ± | 0.34 |
| **TFA** | 14,5 | 55 | 71 | 22 | 0.16 | ± | 0.22 | 0.16 | ± | 0.21 | 0.16 | ± | 0.27 | 0.12 | ± | 0.22 | 0.18 | ± | 0.27 | 0.18 | ± | 0.26 |
|  |  |  |  |  |  |  |  |  |  |  |  |  |  |  |  |  |  |  |  |  |  |  |

Method as listed in Table 1. Nr of cases: for left (L), right(R) and surrogate data (S). Data for repeated measurements (T1 and T2, mean ± SD)

**Table S2(g).** Coherence VLF results per method.

|  |  |  |  |  |  |  |  |  |  |  |  |  |  |  |  |  |  |  |  |  |  |  |
| --- | --- | --- | --- | --- | --- | --- | --- | --- | --- | --- | --- | --- | --- | --- | --- | --- | --- | --- | --- | --- | --- | --- |
|  |  | nr of cases | | | L | | | | | | R | | | | | | S | | | | | |
| Coherence VLF | Method | L | R | S | T1 | | | T2 | | | T1 | | | T2 | | | T1 | | | T2 | | |
| **TFA** | 1,1 | 54 | 68 | 22 | 0.52 | ± | 0.11 | 0.50 | ± | 0.13 | 0.50 | ± | 0.13 | 0.50 | ± | 0.13 | 0.45 | ± | 0.10 | 0.45 | ± | 0.08 |
| **TFA** | 3,1 | 55 | 71 | 22 | 0.52 | ± | 0.15 | 0.50 | ± | 0.16 | 0.50 | ± | 0.15 | 0.50 | ± | 0.16 | 0.41 | ± | 0.13 | 0.44 | ± | 0.07 |
| **TFA** | 5,1 | 55 | 71 | 22 | 0.50 | ± | 0.16 | 0.47 | ± | 0.16 | 0.47 | ± | 0.16 | 0.48 | ± | 0.16 | 0.36 | ± | 0.12 | 0.39 | ± | 0.08 |
| **TFA** | 6,1 | 55 | 71 | 22 | 0.54 | ± | 0.14 | 0.50 | ± | 0.14 | 0.51 | ± | 0.14 | 0.51 | ± | 0.15 | 0.45 | ± | 0.11 | 0.47 | ± | 0.09 |
| **TFA** | 7,1 | 55 | 71 | 22 | 0.40 | ± | 0.15 | 0.35 | ± | 0.16 | 0.37 | ± | 0.16 | 0.36 | ± | 0.16 | 0.28 | ± | 0.10 | 0.30 | ± | 0.11 |
| **Wavelets** | 8,2 | 55 | 71 | 22 | 0.54 | ± | 0.15 | 0.50 | ± | 0.15 | 0.53 | ± | 0.14 | 0.51 | ± | 0.15 | 0.46 | ± | 0.12 | 0.47 | ± | 0.11 |
| **TFA** | 9,1 | 55 | 71 | 22 | 0.72 | ± | 0.12 | 0.70 | ± | 0.12 | 0.70 | ± | 0.11 | 0.71 | ± | 0.11 | 0.64 | ± | 0.08 | 0.64 | ± | 0.07 |
| **TFA** | 11,1 | 55 | 71 | 22 | 0.40 | ± | 0.18 | 0.35 | ± | 0.17 | 0.37 | ± | 0.17 | 0.38 | ± | 0.18 | 0.26 | ± | 0.13 | 0.30 | ± | 0.10 |
| **TFA** | 11,2 | 55 | 71 | 22 | 0.40 | ± | 0.18 | 0.35 | ± | 0.17 | 0.37 | ± | 0.17 | 0.38 | ± | 0.18 | 0.26 | ± | 0.13 | 0.30 | ± | 0.10 |
| **TFA** | 12,1 | 55 | 71 | 22 | 0.43 | ± | 0.16 | 0.39 | ± | 0.16 | 0.40 | ± | 0.15 | 0.41 | ± | 0.16 | 0.30 | ± | 0.12 | 0.35 | ± | 0.09 |
| **TFA** | 13,1 | 55 | 71 | 22 | 0.54 | ± | 0.19 | 0.52 | ± | 0.20 | 0.51 | ± | 0.16 | 0.52 | ± | 0.20 | 0.56 | ± | 0.20 | 0.56 | ± | 0.21 |
| **TFA** | 14,5 | 55 | 71 | 22 | 0.49 | ± | 0.17 | 0.47 | ± | 0.16 | 0.48 | ± | 0.16 | 0.50 | ± | 0.16 | 0.36 | ± | 0.14 | 0.39 | ± | 0.10 |
|  |  |  |  |  |  |  |  |  |  |  |  |  |  |  |  |  |  |  |  |  |  |  |

Method as listed in Table 1. Nr of cases: for left (L), right(R) and surrogate data (S). Data for repeated measurements (T1 and T2, mean ± SD)

**Table S2(h).** Coherence LF results per method.

|  |  |  |  |  |  |  |  |  |  |  |  |  |  |  |  |  |  |  |  |  |  |  |
| --- | --- | --- | --- | --- | --- | --- | --- | --- | --- | --- | --- | --- | --- | --- | --- | --- | --- | --- | --- | --- | --- | --- |
|  |  | nr of cases | | | L | | | | | | R | | | | | | S | | | | | |
| Coherence LF | Method | L | R | S | T1 | | | T2 | | | T1 | | | T2 | | | T1 | | | T2 | | |
| **TFA** | 1,1 | 54 | 68 | 22 | 0.74 | ± | 0.15 | 0.68 | ± | 0.15 | 0.72 | ± | 0.16 | 0.69 | ± | 0.14 | 0.91 | ± | 0.12 | 0.91 | ± | 0.10 |
| **TFA** | 3,1 | 55 | 71 | 22 | 0.73 | ± | 0.14 | 0.70 | ± | 0.13 | 0.69 | ± | 0.15 | 0.70 | ± | 0.13 | 0.82 | ± | 0.17 | 0.87 | ± | 0.11 |
| **TFA** | 5,1 | 55 | 71 | 22 | 0.75 | ± | 0.15 | 0.72 | ± | 0.15 | 0.71 | ± | 0.17 | 0.69 | ± | 0.15 | 0.90 | ± | 0.13 | 0.90 | ± | 0.08 |
| **TFA** | 6,1 | 55 | 71 | 22 | 0.75 | ± | 0.14 | 0.71 | ± | 0.15 | 0.70 | ± | 0.17 | 0.68 | ± | 0.15 | 0.87 | ± | 0.12 | 0.87 | ± | 0.08 |
| **TFA** | 7,1 | 55 | 71 | 22 | 0.70 | ± | 0.16 | 0.67 | ± | 0.16 | 0.65 | ± | 0.19 | 0.64 | ± | 0.16 | 0.78 | ± | 0.11 | 0.79 | ± | 0.09 |
| **Wavelets** | 8,2 | 55 | 71 | 22 | 0.75 | ± | 0.13 | 0.73 | ± | 0.12 | 0.72 | ± | 0.15 | 0.70 | ± | 0.12 | 0.78 | ± | 0.10 | 0.77 | ± | 0.10 |
| **TFA** | 9,1 | 55 | 71 | 22 | 0.84 | ± | 0.11 | 0.82 | ± | 0.10 | 0.81 | ± | 0.12 | 0.80 | ± | 0.10 | 0.90 | ± | 0.08 | 0.91 | ± | 0.05 |
| **TFA** | 11,1 | 55 | 71 | 22 | 0.71 | ± | 0.16 | 0.68 | ± | 0.15 | 0.67 | ± | 0.17 | 0.66 | ± | 0.15 | 0.87 | ± | 0.14 | 0.87 | ± | 0.10 |
| **TFA** | 11,2 | 55 | 71 | 22 | 0.71 | ± | 0.16 | 0.68 | ± | 0.15 | 0.67 | ± | 0.17 | 0.66 | ± | 0.15 | 0.87 | ± | 0.14 | 0.87 | ± | 0.10 |
| **TFA** | 12,1 | 55 | 71 | 22 | 0.75 | ± | 0.14 | 0.71 | ± | 0.15 | 0.70 | ± | 0.16 | 0.68 | ± | 0.14 | 0.85 | ± | 0.11 | 0.85 | ± | 0.08 |
| **TFA** | 13,1 | 55 | 71 | 22 | 0.75 | ± | 0.12 | 0.71 | ± | 0.14 | 0.73 | ± | 0.13 | 0.69 | ± | 0.14 | 0.83 | ± | 0.13 | 0.82 | ± | 0.14 |
| **TFA** | 14,5 | 55 | 71 | 22 | 0.75 | ± | 0.15 | 0.72 | ± | 0.15 | 0.71 | ± | 0.17 | 0.70 | ± | 0.14 | 0.87 | ± | 0.12 | 0.88 | ± | 0.06 |
|  |  |  |  |  |  |  |  |  |  |  |  |  |  |  |  |  |  |  |  |  |  |  |

Method as listed in Table 1. Nr of cases: for left (L), right(R) and surrogate data (S). Data for repeated measurements (T1 and T2, mean ± SD)

**Table S2(i).** Coherence HF results per method.

|  |  |  |  |  |  |  |  |  |  |  |  |  |  |  |  |  |  |  |  |  |  |  |
| --- | --- | --- | --- | --- | --- | --- | --- | --- | --- | --- | --- | --- | --- | --- | --- | --- | --- | --- | --- | --- | --- | --- |
|  |  | nr of cases | | | L | | | | | | R | | | | | | S | | | | | |
| Coherence HF | Method | L | R | S | T1 | | | T2 | | | T1 | | | T2 | | | T1 | | | T2 | | |
| **TFA** | 1,1 | 54 | 68 | 22 | 0.65 | ± | 0.15 | 0.62 | ± | 0.17 | 0.62 | ± | 0.17 | 0.63 | ± | 0.18 | 0.98 | ± | 0.02 | 0.98 | ± | 0.02 |
| **TFA** | 3,1 | 55 | 71 | 22 | 0.71 | ± | 0.14 | 0.68 | ± | 0.15 | 0.68 | ± | 0.16 | 0.68 | ± | 0.16 | 0.92 | ± | 0.13 | 0.93 | ± | 0.09 |
| **TFA** | 5,1 | 55 | 71 | 22 | 0.68 | ± | 0.14 | 0.63 | ± | 0.16 | 0.63 | ± | 0.16 | 0.64 | ± | 0.16 | 0.98 | ± | 0.08 | 0.99 | ± | 0.05 |
| **TFA** | 6,1 | 55 | 71 | 22 | 0.67 | ± | 0.14 | 0.63 | ± | 0.15 | 0.63 | ± | 0.16 | 0.64 | ± | 0.15 | 0.97 | ± | 0.08 | 0.98 | ± | 0.05 |
| **TFA** | 7,1 | 55 | 71 | 22 | 0.66 | ± | 0.16 | 0.61 | ± | 0.17 | 0.61 | ± | 0.19 | 0.62 | ± | 0.18 | 0.98 | ± | 0.06 | 0.98 | ± | 0.06 |
| **Wavelets** | 8,2 | 55 | 71 | 22 | 0.76 | ± | 0.13 | 0.74 | ± | 0.14 | 0.73 | ± | 0.15 | 0.74 | ± | 0.13 | 0.96 | ± | 0.04 | 0.96 | ± | 0.04 |
| **TFA** | 9,1 | 55 | 71 | 22 | 0.80 | ± | 0.10 | 0.76 | ± | 0.12 | 0.76 | ± | 0.12 | 0.77 | ± | 0.12 | 0.99 | ± | 0.05 | 0.99 | ± | 0.04 |
| **TFA** | 11,1 | 55 | 71 | 22 | 0.58 | ± | 0.16 | 0.55 | ± | 0.18 | 0.55 | ± | 0.18 | 0.56 | ± | 0.18 | 0.99 | ± | 0.06 | 0.99 | ± | 0.04 |
| **TFA** | 11,2 | 55 | 71 | 22 | 0.58 | ± | 0.16 | 0.55 | ± | 0.18 | 0.55 | ± | 0.18 | 0.56 | ± | 0.18 | 0.99 | ± | 0.06 | 0.99 | ± | 0.04 |
| **TFA** | 12,1 | 55 | 71 | 22 | 0.74 | ± | 0.16 | 0.69 | ± | 0.16 | 0.71 | ± | 0.17 | 0.70 | ± | 0.15 | 0.98 | ± | 0.10 | 0.98 | ± | 0.08 |
| **TFA** | 13,1 | 55 | 71 | 22 | 0.76 | ± | 0.09 | 0.74 | ± | 0.11 | 0.75 | ± | 0.09 | 0.76 | ± | 0.10 | 0.93 | ± | 0.03 | 0.94 | ± | 0.03 |
| **TFA** | 14,5 | 55 | 71 | 22 | 0.68 | ± | 0.13 | 0.63 | ± | 0.16 | 0.64 | ± | 0.15 | 0.66 | ± | 0.15 | 0.98 | ± | 0.08 | 0.99 | ± | 0.04 |
|  |  |  |  |  |  |  |  |  |  |  |  |  |  |  |  |  |  |  |  |  |  |  |

Method as listed in Table 1. Nr of cases: for left (L), right(R) and surrogate data (S). Data for repeated measurements (T1 and T2, mean ± SD)

**Table S2(j).** ARI-like results per method.

|  |  |  |  |  |  |  |  |  |  |  |  |  |  |  |  |  |  |  |  |  |  |  |
| --- | --- | --- | --- | --- | --- | --- | --- | --- | --- | --- | --- | --- | --- | --- | --- | --- | --- | --- | --- | --- | --- | --- |
|  |  | nr of cases | | | L | | | | | | R | | | | | | S | | | | | |
| ARI | Method | L | R | S | T1 | | | T2 | | | T1 | | | T2 | | | T1 | | | T2 | | |
| **ARI** | 1,2 | 54 | 68 | 22 | 5.30 | ± | 1.67 | 5.63 | ± | 1.32 | 5.53 | ± | 1.66 | 5.27 | ± | 1.56 | 4.64 | ± | 2.54 | 4.44 | ± | 2.68 |
| **ARI** | 4,1 | 55 | 71 | 22 | 5.38 | ± | 1.64 | 5.56 | ± | 1.32 | 5.48 | ± | 1.73 | 5.34 | ± | 1.47 | 4.55 | ± | 2.70 | 4.47 | ± | 2.81 |
| **ARMA-ARI/ARX** | 4,2 | 55 | 71 | 22 | 5.30 | ± | 1.69 | 5.80 | ± | 1.55 | 5.38 | ± | 1.80 | 5.48 | ± | 1.63 | 4.33 | ± | 2.94 | 4.33 | ± | 3.05 |
| **ARMA-ARI/ARX** | 4,3 | 55 | 71 | 22 | 5.43 | ± | 1.54 | 6.02 | ± | 1.36 | 5.49 | ± | 1.62 | 5.81 | ± | 1.50 | 4.44 | ± | 2.84 | 4.24 | ± | 3.11 |
| **ARMA-ARI/ARX** | 8,1 | 55 | 71 | 22 | 14.21 | ± | 5.11 | 13.30 | ± | 6.51 | 13.66 | ± | 6.88 | 15.46 | ± | 5.50 | 16.00 | ± | 16.50 | 19.33 | ± | 18.59 |
| **IR-filter** | 11,5 | 55 | 71 | 22 | -1.06 | ± | 0.56 | -1.01 | ± | 0.46 | -1.06 | ± | 0.52 | -1.03 | ± | 0.42 | -0.26 | ± | 0.22 | -0.26 | ± | 0.22 |
| **ARI** | 12,2 | 55 | 71 | 22 | 5.55 | ± | 2.39 | 5.95 | ± | 2.05 | 5.54 | ± | 2.44 | 5.69 | ± | 2.23 | 4.59 | ± | 3.14 | 4.65 | ± | 3.07 |
|  |  |  |  |  |  |  |  |  |  |  |  |  |  |  |  |  |  |  |  |  |  |  |

Method as listed in Table 1. Nr of cases: for left (L), right(R) and surrogate data (S). Data for repeated measurements (T1 and T2, mean ± SD)

**Table S2(k).** Correletion-like results per method.

|  |  |  |  |  |  |  |  |  |  |  |  |  |  |  |  |  |  |  |  |  |  |  |
| --- | --- | --- | --- | --- | --- | --- | --- | --- | --- | --- | --- | --- | --- | --- | --- | --- | --- | --- | --- | --- | --- | --- |
|  |  | nr of cases | | | L | | | | | | R | | | | | | S | | | | | |
| Correlation | Method | L | R | S | T1 | | | T2 | | | T1 | | | T2 | | | T1 | | | T2 | | |
| **Correlation coefficient** | 5,2 | 55 | 71 | 22 | 0.44 | ± | 0.14 | 0.39 | ± | 0.13 | 0.40 | ± | 0.14 | 0.38 | ± | 0.14 | 0.36 | ± | 0.06 | 0.36 | ± | 0.09 |
| **Correlation coefficient** | 9,2 | 55 | 71 | 22 | 0.47 | ± | 0.14 | 0.45 | ± | 0.17 | 0.45 | ± | 0.15 | 0.45 | ± | 0.16 | 0.44 | ± | 0.16 | 0.47 | ± | 0.14 |

Methodas listed in Table 1. Nr of cases: for left (L), right(R) and surrogate data (S). Data for repeated measurements (T1 and T2, mean ± SD)

**Table S3(a)**. Bland Altman results for TFA-like methods.

|  |  |  | **nr of cases** | | | **INT** | | | **bias** | | | **ULOA** | | | **LLOA limit** | | |  |
| --- | --- | --- | --- | --- | --- | --- | --- | --- | --- | --- | --- | --- | --- | --- | --- | --- | --- | --- |
| **TFA-like** | **Method** | **Variable** | **R** | **L** | **S** | **R** | **L** | **S** | **R** | **L** | **S** | **R** | **L** | **S** | **R** | **L** | **S** |  |
| TFA | 1,1 | Gain_LF | 53 | 67 | 22 | 0.74 | 0.83 | 0.08 | 0.08 | 0.07 | -0.01 | 0.82 | 0.90 | 0.07 | -0.66 | -0.76 | -0.09 |  |
|  | 1,1 | Gain_VLF | 53 | 67 | 22 | 0.73 | 0.80 | 0.11 | 0.11 | 0.06 | -0.01 | 0.84 | 0.86 | 0.10 | -0.62 | -0.74 | -0.13 |  |
|  | 1,1 | Phase_LF | 53 | 67 | 22 | 0.48 | 0.51 | 0.06 | -0.01 | 0.00 | 0.00 | 0.47 | 0.51 | 0.06 | -0.50 | -0.51 | -0.06 |  |
|  | 1,1 | Phase_VLF | 53 | 67 | 22 | 0.86 | 0.93 | 0.83 | -0.01 | 0.08 | -0.06 | 0.85 | 1.01 | 0.77 | -0.87 | -0.85 | -0.89 |  |
|  | 3,1 | Gain_LF | 55 | 71 | 22 | 0.91 | 0.92 | 0.09 | 0.08 | 0.07 | -0.01 | 0.99 | 0.98 | 0.07 | -0.83 | -0.85 | -0.10 |  |
|  | 3,1 | Gain_VLF | 55 | 71 | 22 | 0.66 | 0.69 | 0.21 | 0.07 | 0.02 | -0.02 | 0.73 | 0.70 | 0.19 | -0.59 | -0.67 | -0.23 |  |
|  | 3,1 | Phase_LF | 55 | 71 | 22 | 0.42 | 0.53 | 0.22 | -0.01 | 0.01 | 0.02 | 0.41 | 0.54 | 0.24 | -0.43 | -0.52 | -0.20 |  |
|  | 3,1 | Phase_VLF | 55 | 71 | 22 | 1.03 | 1.09 | 1.32 | -0.02 | 0.02 | 0.08 | 1.01 | 1.11 | 1.40 | -1.04 | -1.07 | -1.24 |  |
|  | 3,2 | Gain_LF | 55 | 71 | 22 | 1.38 | 1.22 | 0.11 | 0.13 | 0.08 | -0.02 | 1.51 | 1.30 | 0.09 | -1.26 | -1.13 | -0.13 |  |
|  | 3,2 | Gain_VLF | 55 | 71 | 22 | 1.02 | 1.06 | 0.42 | 0.12 | 0.00 | -0.02 | 1.14 | 1.06 | 0.40 | -0.91 | -1.06 | -0.45 |  |
|  | 5,1 | Gain_LF | 55 | 71 | 22 | 0.83 | 0.90 | 0.09 | 0.07 | 0.08 | -0.01 | 0.90 | 0.98 | 0.08 | -0.75 | -0.81 | -0.10 |  |
|  | 5,1 | Gain_VLF | 55 | 71 | 22 | 0.72 | 0.78 | 0.20 | 0.08 | 0.01 | -0.02 | 0.81 | 0.79 | 0.18 | -0.64 | -0.77 | -0.22 |  |
|  | 5,1 | Phase_LF | 55 | 71 | 22 | 0.55 | 0.71 | 0.20 | 0.00 | 0.05 | 0.00 | 0.55 | 0.76 | 0.19 | -0.55 | -0.66 | -0.20 |  |
|  | 5,1 | Phase_VLF | 55 | 71 | 22 | 1.44 | 1.39 | 1.41 | -0.06 | 0.00 | 0.02 | 1.38 | 1.40 | 1.43 | -1.49 | -1.39 | -1.38 |  |
|  | 6,1 | Gain_LF | 55 | 71 | 22 | 0.53 | 0.62 | 0.09 | 0.06 | 0.07 | -0.01 | 0.59 | 0.69 | 0.08 | -0.47 | -0.55 | -0.10 |  |
|  | 6,1 | Gain_VLF | 55 | 71 | 22 | 0.42 | 0.48 | 0.20 | 0.05 | -0.01 | -0.01 | 0.47 | 0.47 | 0.19 | -0.37 | -0.49 | -0.21 |  |
|  | 6,1 | Phase_LF | 55 | 71 | 22 | 0.42 | 0.56 | 0.23 | -0.03 | -0.02 | 0.03 | 0.39 | 0.54 | 0.26 | -0.45 | -0.58 | -0.20 |  |
|  | 6,1 | Phase_VLF | 55 | 71 | 22 | 0.67 | 0.74 | 0.63 | -0.02 | 0.00 | -0.02 | 0.66 | 0.74 | 0.61 | -0.69 | -0.75 | -0.65 |  |
|  | 7,1 | Gain_LF | 55 | 71 | 22 | 0.83 | 0.87 | 0.10 | 0.08 | 0.06 | -0.01 | 0.91 | 0.94 | 0.08 | -0.74 | -0.81 | -0.11 |  |
|  | 7,1 | Gain_VLF | 55 | 71 | 22 | 0.65 | 0.76 | 0.15 | 0.11 | 0.03 | -0.02 | 0.76 | 0.79 | 0.12 | -0.55 | -0.72 | -0.17 |  |
|  | 7,1 | Phase_LF | 55 | 71 | 22 | 0.43 | 0.48 | 0.23 | -0.02 | -0.02 | 0.01 | 0.41 | 0.46 | 0.24 | -0.45 | -0.49 | -0.22 |  |
|  | 7,1 | Phase_VLF | 55 | 71 | 22 | 1.53 | 2.00 | 1.03 | 0.00 | -0.12 | 0.08 | 1.53 | 1.88 | 1.11 | -1.54 | -2.13 | -0.94 |  |
|  | 9,1 | Gain_LF | 55 | 71 | 22 | 0.79 | 0.87 | 0.09 | 0.07 | 0.08 | -0.01 | 0.87 | 0.94 | 0.08 | -0.72 | -0.79 | -0.10 |  |
|  | 9,1 | Gain_VLF | 55 | 71 | 22 | 0.50 | 0.62 | 0.18 | 0.06 | 0.01 | -0.02 | 0.56 | 0.63 | 0.17 | -0.44 | -0.61 | -0.20 |  |
|  | 9,1 | Phase_LF | 55 | 71 | 22 | 0.42 | 0.53 | 0.20 | -0.02 | -0.03 | 0.01 | 0.40 | 0.50 | 0.20 | -0.44 | -0.56 | -0.19 |  |
|  | 9,1 | Phase_VLF | 55 | 71 | 20 | 0.76 | 0.90 | 0.98 | -0.01 | 0.00 | -0.06 | 0.76 | 0.91 | 0.93 | -0.77 | -0.90 | -1.04 |  |
|  | 11,1 | Gain_LF | 55 | 71 | 22 | 0.80 | 0.87 | 0.08 | 0.07 | 0.07 | -0.01 | 0.87 | 0.93 | 0.07 | -0.73 | -0.80 | -0.09 |  |
|  | 11,1 | Gain_VLF | 55 | 71 | 22 | 0.68 | 0.77 | 0.19 | 0.10 | 0.00 | -0.02 | 0.77 | 0.78 | 0.17 | -0.58 | -0.77 | -0.21 |  |
|  | 11,1 | Phase_LF | 55 | 71 | 22 | 0.39 | 0.47 | 0.15 | -0.01 | 0.01 | 0.01 | 0.38 | 0.48 | 0.16 | -0.40 | -0.46 | -0.14 |  |
|  | 11,1 | Phase_VLF | 55 | 69 | 20 | 1.01 | 1.26 | 0.91 | -0.09 | -0.01 | -0.06 | 0.93 | 1.25 | 0.85 | -1.10 | -1.26 | -0.97 |  |
|  | 11,2 | Gain_LF | 55 | 71 | 22 | 1.22 | 1.15 | 0.10 | 0.11 | 0.09 | -0.01 | 1.33 | 1.23 | 0.09 | -1.11 | -1.06 | -0.12 |  |
|  | 11,2 | Gain_VLF | 55 | 71 | 22 | 1.09 | 1.26 | 0.38 | 0.16 | -0.02 | -0.04 | 1.25 | 1.24 | 0.35 | -0.93 | -1.27 | -0.42 |  |
|  | 11,3 | Gain_LF | 55 | 71 | 22 | 0.85 | 0.83 | 0.06 | 0.12 | 0.09 | 0.00 | 0.98 | 0.92 | 0.06 | -0.73 | -0.74 | -0.06 |  |
|  | 11,3 | Gain_VLF | 55 | 71 | 22 | 0.84 | 0.98 | 0.36 | 0.15 | 0.00 | -0.03 | 0.99 | 0.97 | 0.33 | -0.69 | -0.98 | -0.39 |  |
|  | 12,1 | Gain_LF | 54 | 71 | 22 | 0.86 | 0.95 | 0.08 | 0.08 | 0.07 | -0.01 | 0.94 | 1.03 | 0.08 | -0.78 | -0.88 | -0.09 |  |
|  | 12,1 | Gain_VLF | 37 | 46 | 13 | 1.20 | 1.41 | 0.28 | 0.10 | 0.04 | 0.03 | 1.30 | 1.46 | 0.31 | -1.09 | -1.37 | -0.25 |  |
|  | 12,1 | Phase_LF | 54 | 71 | 22 | 0.45 | 0.63 | 0.23 | -0.03 | -0.04 | 0.00 | 0.41 | 0.59 | 0.24 | -0.48 | -0.67 | -0.23 |  |
|  | 12,1 | Phase_VLF | 36 | 45 | 13 | 1.04 | 1.24 | 1.23 | -0.07 | -0.15 | 0.04 | 0.96 | 1.09 | 1.28 | -1.11 | -1.40 | -1.19 |  |
|  | 13,1 | Gain_LF | 55 | 71 | 22 | 0.56 | 0.64 | 0.14 | 0.06 | 0.08 | 0.00 | 0.62 | 0.72 | 0.14 | -0.50 | -0.56 | -0.14 |  |
|  | 13,1 | Gain_VLF | 55 | 71 | 22 | 0.50 | 0.53 | 0.25 | 0.03 | -0.02 | -0.05 | 0.53 | 0.50 | 0.20 | -0.46 | -0.55 | -0.30 |  |
|  | 13,1 | Phase_LF | 55 | 71 | 22 | 0.58 | 0.56 | 0.44 | 0.01 | -0.01 | -0.03 | 0.59 | 0.55 | 0.41 | -0.56 | -0.56 | -0.47 |  |
|  | 13,1 | Phase_VLF | 55 | 71 | 22 | 1.41 | 1.47 | 1.40 | 0.12 | 0.01 | -0.19 | 1.52 | 1.48 | 1.21 | -1.29 | -1.47 | -1.60 |  |
|  | 14,5 | Gain_LF | 55 | 71 | 22 | 0.80 | 0.91 | 0.10 | 0.07 | 0.07 | -0.01 | 0.86 | 0.99 | 0.09 | -0.73 | -0.84 | -0.11 |  |
|  | 14,5 | Gain_VLF | 55 | 71 | 22 | 0.76 | 0.84 | 0.24 | 0.07 | 0.00 | -0.03 | 0.82 | 0.84 | 0.21 | -0.69 | -0.83 | -0.28 |  |
|  | 14,5 | Phase_LF | 55 | 71 | 22 | 0.53 | 0.65 | 0.24 | -0.02 | -0.04 | 0.02 | 0.51 | 0.61 | 0.25 | -0.55 | -0.69 | -0.22 |  |
|  | 14,5 | Phase_VLF | 55 | 71 | 22 | 1.33 | 1.35 | 1.54 | 0.05 | -0.01 | 0.11 | 1.38 | 1.34 | 1.65 | -1.27 | -1.35 | -1.43 |  |
|  |  |  |  |  |  |  |  |  |  |  |  |  |  |  |  |  |  |  |
| Laguerre | 2,1 | Gain_HF | 55 | 71 | 22 | 1.05 | 1.30 | 0.15 | 0.17 | 0.04 | -0.02 | 1.22 | 1.34 | 0.13 | -0.88 | -1.26 | -0.17 |  |
|  | 2,1 | Gain_LF | 55 | 71 | 22 | 0.60 | 0.81 | 0.13 | 0.08 | 0.02 | -0.01 | 0.68 | 0.83 | 0.12 | -0.52 | -0.79 | -0.15 |  |
|  | 2,1 | Gain_VLF | 55 | 71 | 22 | 0.55 | 0.61 | 0.09 | 0.10 | 0.03 | -0.01 | 0.65 | 0.64 | 0.08 | -0.45 | -0.58 | -0.10 |  |
|  | 2,1 | Phase_HF | 55 | 71 | 22 | 0.54 | 0.68 | 0.36 | 0.04 | 0.01 | -0.01 | 0.58 | 0.69 | 0.36 | -0.50 | -0.68 | -0.37 |  |
|  | 2,1 | Phase_LF | 55 | 71 | 22 | 0.60 | 0.64 | 0.38 | -0.05 | -0.01 | 0.02 | 0.55 | 0.63 | 0.40 | -0.64 | -0.65 | -0.36 |  |
|  | 2,1 | Phase_VLF | 55 | 71 | 22 | 1.36 | 1.49 | 0.79 | -0.16 | -0.04 | 0.03 | 1.20 | 1.45 | 0.82 | -1.52 | -1.53 | -0.76 |  |
|  | 2,2 | Gain_HF | 55 | 71 | 22 | 0.99 | 1.18 | 0.15 | 0.16 | 0.06 | -0.01 | 1.15 | 1.24 | 0.14 | -0.83 | -1.12 | -0.17 |  |
|  | 2,2 | Gain_LF | 55 | 71 | 22 | 0.63 | 0.69 | 0.14 | 0.09 | 0.04 | -0.01 | 0.73 | 0.73 | 0.13 | -0.54 | -0.65 | -0.15 |  |
|  | 2,2 | Gain_VLF | 55 | 71 | 22 | 0.44 | 0.51 | 0.09 | 0.09 | 0.04 | -0.01 | 0.53 | 0.55 | 0.08 | -0.35 | -0.47 | -0.10 |  |
|  | 2,2 | Phase_HF | 55 | 71 | 22 | 0.51 | 0.62 | 0.35 | 0.03 | 0.01 | -0.01 | 0.54 | 0.63 | 0.35 | -0.48 | -0.61 | -0.36 |  |
|  | 2,2 | Phase_LF | 55 | 71 | 22 | 0.56 | 0.64 | 0.41 | -0.04 | 0.00 | 0.01 | 0.53 | 0.63 | 0.43 | -0.60 | -0.64 | -0.40 |  |
|  | 2,2 | Phase_VLF | 55 | 71 | 22 | 1.34 | 1.52 | 0.76 | -0.10 | -0.04 | 0.02 | 1.24 | 1.47 | 0.78 | -1.43 | -1.56 | -0.75 |  |
|  | 14,3 | Gain_HF | 55 | 71 | 22 | 1.16 | 1.26 | 0.17 | 0.10 | 0.01 | -0.01 | 1.26 | 1.27 | 0.16 | -1.07 | -1.25 | -0.17 |  |
|  | 14,3 | Gain_LF | 55 | 71 | 22 | 0.93 | 0.94 | 0.19 | 0.10 | 0.06 | -0.01 | 1.03 | 0.99 | 0.18 | -0.82 | -0.88 | -0.19 |  |
|  | 14,3 | Gain_VLF | 55 | 71 | 22 | 0.57 | 0.58 | 0.17 | 0.06 | -0.02 | -0.02 | 0.63 | 0.56 | 0.15 | -0.51 | -0.59 | -0.19 |  |
|  | 14,3 | Phase_HF | 55 | 71 | 22 | 1.06 | 0.90 | 0.46 | 0.08 | 0.00 | 0.03 | 1.14 | 0.90 | 0.49 | -0.98 | -0.89 | -0.43 |  |
|  | 14,3 | Phase_LF | 55 | 71 | 22 | 0.96 | 1.01 | 0.77 | 0.04 | -0.08 | 0.05 | 1.00 | 0.93 | 0.82 | -0.92 | -1.08 | -0.73 |  |
|  | 14,3 | Phase_VLF | 55 | 71 | 22 | 1.41 | 1.55 | 0.99 | -0.03 | -0.09 | 0.12 | 1.38 | 1.47 | 1.11 | -1.44 | -1.64 | -0.87 |  |
|  | 14,4 | Gain_HF | 55 | 71 | 22 | 1.20 | 1.23 | 0.19 | 0.16 | 0.06 | -0.01 | 1.36 | 1.28 | 0.17 | -1.04 | -1.17 | -0.20 |  |
|  | 14,4 | Gain_LF | 55 | 71 | 22 | 0.91 | 0.89 | 0.19 | 0.13 | 0.09 | -0.01 | 1.04 | 0.99 | 0.18 | -0.78 | -0.80 | -0.20 |  |
|  | 14,4 | Gain_VLF | 55 | 71 | 22 | 0.69 | 0.56 | 0.17 | 0.06 | 0.01 | -0.04 | 0.75 | 0.57 | 0.13 | -0.63 | -0.55 | -0.20 |  |
|  | 14,4 | Phase_HF | 55 | 71 | 22 | 0.89 | 1.10 | 0.50 | 0.06 | -0.08 | 0.02 | 0.95 | 1.01 | 0.52 | -0.83 | -1.18 | -0.48 |  |
|  | 14,4 | Phase_LF | 55 | 71 | 22 | 0.88 | 1.02 | 0.67 | 0.05 | -0.06 | -0.03 | 0.93 | 0.96 | 0.64 | -0.84 | -1.07 | -0.70 |  |
|  | 14,4 | Phase_VLF | 55 | 71 | 22 | 1.32 | 1.43 | 0.87 | -0.07 | -0.12 | 0.08 | 1.25 | 1.31 | 0.95 | -1.40 | -1.55 | -0.79 |  |
|  |  |  |  |  |  |  |  |  |  |  |  |  |  |  |  |  |  |  |
| Wavelet | 8,2 | Coh_HF | 55 | 71 | 22 | 0.19 | 0.19 | 0.03 | 0.02 | -0.01 | 0.00 | 0.21 | 0.18 | 0.03 | -0.18 | -0.21 | -0.03 |  |
|  | 8,2 | Coh_LF | 55 | 71 | 22 | 0.21 | 0.23 | 0.14 | 0.03 | 0.02 | 0.02 | 0.24 | 0.25 | 0.16 | -0.18 | -0.22 | -0.12 |  |
|  | 8,2 | Coh_VLF | 55 | 71 | 22 | 0.26 | 0.32 | 0.32 | 0.04 | 0.02 | 0.00 | 0.30 | 0.33 | 0.32 | -0.22 | -0.30 | -0.32 |  |
|  | 8,2 | phase_HF | 55 | 71 | 22 | 0.56 | 0.59 | 0.05 | 0.03 | 0.03 | -0.01 | 0.60 | 0.62 | 0.04 | -0.53 | -0.57 | -0.06 |  |
|  | 8,2 | Phase_LF | 55 | 71 | 22 | 0.45 | 0.49 | 0.22 | -0.03 | -0.02 | 0.00 | 0.42 | 0.47 | 0.22 | -0.48 | -0.51 | -0.22 |  |
|  | 8,2 | Phase_VLF | 55 | 71 | 22 | 1.39 | 1.82 | 1.42 | -0.07 | -0.19 | 0.03 | 1.32 | 1.63 | 1.45 | -1.46 | -2.00 | -1.38 |  |
|  | 12,3 | Gain_HF | 55 | 71 | 22 | 0.92 | 1.00 | 0.44 | 0.03 | 0.04 | 0.03 | 0.95 | 1.04 | 0.47 | -0.89 | -0.96 | -0.41 |  |
|  | 12,3 | Gain_LF | 55 | 71 | 22 | 0.91 | 1.04 | 0.42 | 0.08 | 0.08 | 0.03 | 0.99 | 1.12 | 0.45 | -0.84 | -0.96 | -0.39 |  |
|  | 12,3 | Gain_VLF | 51 | 62 | 16 | 1.03 | 1.14 | 0.33 | 0.12 | -0.03 | -0.03 | 1.15 | 1.10 | 0.30 | -0.92 | -1.17 | -0.36 |  |
|  | 12,3 | Phase_HF | 55 | 71 | 22 | 0.46 | 0.63 | 0.12 | -0.03 | -0.01 | 0.00 | 0.43 | 0.62 | 0.12 | -0.48 | -0.64 | -0.12 |  |
|  | 12,3 | Phase_LF | 55 | 71 | 22 | 0.68 | 0.68 | 0.70 | -0.06 | -0.07 | 0.07 | 0.63 | 0.61 | 0.77 | -0.74 | -0.75 | -0.63 |  |
|  | 12,3 | Phase_VLF | 51 | 62 | 16 | 1.33 | 1.41 | 1.65 | -0.20 | -0.08 | 0.04 | 1.13 | 1.33 | 1.69 | -1.52 | -1.49 | -1.61 |  |
|  |  |  |  |  |  |  |  |  |  |  |  |  |  |  |  |  |  |  |
| IR-filter | 11,4 | Gain_LF | 55 | 71 | 22 | 1.02 | 1.03 | 0.24 | 0.19 | 0.06 | -0.02 | 1.20 | 1.09 | 0.23 | -0.83 | -0.97 | -0.26 |  |
|  | 11,4 | Phase_HF | 54 | 69 | 22 | 0.58 | 0.69 | 0.01 | -0.01 | -0.02 | 0.00 | 0.57 | 0.68 | 0.01 | -0.59 | -0.71 | -0.01 |  |
|  | 11,4 | Phase_LF | 55 | 71 | 22 | 0.39 | 0.47 | 0.15 | -0.01 | 0.01 | 0.01 | 0.38 | 0.48 | 0.16 | -0.40 | -0.46 | -0.14 |  |
|  | 11,4 | Phase_LF | 55 | 71 | 21 | 0.41 | 0.51 | 0.32 | -0.05 | -0.01 | 0.01 | 0.36 | 0.49 | 0.33 | -0.45 | -0.52 | -0.31 |  |
|  | 11,4 | Phase_VLF | 55 | 69 | 20 | 1.01 | 1.26 | 0.91 | -0.09 | -0.01 | -0.06 | 0.93 | 1.25 | 0.85 | -1.10 | -1.26 | -0.97 |  |
|  | 11,6 | Gain_LF | 55 | 71 | 22 | 0.96 | 0.96 | 0.30 | 0.20 | 0.06 | -0.03 | 1.16 | 1.02 | 0.28 | -0.77 | -0.91 | -0.33 |  |
|  | 11,6 | Phase_LF | 55 | 71 | 21 | 0.41 | 0.50 | 0.34 | -0.04 | 0.00 | -0.01 | 0.36 | 0.50 | 0.33 | -0.45 | -0.50 | -0.36 |  |
|  |  |  |  |  |  |  |  |  |  |  |  |  |  |  |  |  |  |  |
| ARX | 14,1 | Gain_HF | 55 | 71 | 22 | 0.67 | 0.74 | 0.08 | 0.09 | 0.03 | -0.01 | 0.76 | 0.77 | 0.07 | -0.58 | -0.70 | -0.09 |  |
|  | 14,1 | Gain_LF | 55 | 71 | 22 | 0.58 | 0.68 | 0.08 | 0.07 | 0.05 | -0.01 | 0.65 | 0.73 | 0.07 | -0.51 | -0.63 | -0.09 |  |
|  | 14,1 | Gain_VLF | 55 | 71 | 22 | 0.53 | 0.68 | 0.13 | 0.06 | 0.02 | 0.00 | 0.59 | 0.69 | 0.13 | -0.48 | -0.66 | -0.14 |  |
|  | 14,1 | Phase_HF | 55 | 71 | 22 | 0.44 | 0.39 | 0.04 | 0.02 | -0.01 | 0.00 | 0.46 | 0.38 | 0.05 | -0.42 | -0.39 | -0.04 |  |
|  | 14,1 | Phase_LF | 55 | 71 | 22 | 0.69 | 0.57 | 0.14 | -0.01 | -0.01 | -0.01 | 0.68 | 0.56 | 0.13 | -0.70 | -0.59 | -0.15 |  |
|  | 14,1 | Phase_VLF | 55 | 71 | 22 | 0.96 | 1.19 | 0.85 | -0.04 | 0.01 | 0.05 | 0.92 | 1.19 | 0.90 | -1.00 | -1.18 | -0.79 |  |
|  | 14,2 | Gain_HF | 55 | 71 | 22 | 0.66 | 0.75 | 0.08 | 0.09 | 0.03 | -0.01 | 0.75 | 0.79 | 0.07 | -0.57 | -0.72 | -0.09 |  |
|  | 14,2 | Gain_LF | 55 | 71 | 22 | 0.57 | 0.69 | 0.08 | 0.08 | 0.04 | -0.01 | 0.65 | 0.74 | 0.07 | -0.49 | -0.65 | -0.09 |  |
|  | 14,2 | Gain_VLF | 55 | 71 | 22 | 0.61 | 0.64 | 0.15 | 0.06 | 0.04 | 0.00 | 0.67 | 0.68 | 0.14 | -0.54 | -0.60 | -0.15 |  |
|  | 14,2 | Phase_HF | 55 | 71 | 22 | 0.54 | 0.61 | 0.04 | 0.03 | 0.01 | 0.00 | 0.56 | 0.62 | 0.04 | -0.51 | -0.59 | -0.04 |  |
|  | 14,2 | Phase_LF | 55 | 71 | 22 | 0.53 | 0.62 | 0.15 | -0.01 | -0.01 | -0.01 | 0.51 | 0.62 | 0.13 | -0.54 | -0.63 | -0.16 |  |
|  | 14,2 | Phase_VLF | 55 | 71 | 22 | 0.99 | 1.06 | 1.15 | 0.00 | -0.03 | 0.14 | 1.00 | 1.03 | 1.28 | -0.99 | -1.09 | -1.01 |  |

Results of individual methods per method group. Methods and units are listed in Table 1. T1: measurement 1; T2: measurement 2; bias: T1-T2; INT: interval (=1.96*SD_bias_); ULOA: upper limit of agreement (=mean_bias_-interval); LLOA: lower limit of agreement (=mean_bias_+interval);

**Table S3(b)**. Bland Altman results for ARI-like methods.

|  |  |  |  | |  | |  |  | |  | |  | |  |  |  |  |  |  |  |  |  |  |
| --- | --- | --- | --- | --- | --- | --- | --- | --- | --- | --- | --- | --- | --- | --- | --- | --- | --- | --- | --- | --- | --- | --- | --- |
|  |  |  | **nr of cases** | | | | | **LOA** | | | | | | **mean T1 T2** | | | **upper limit** | | | **lower limit** | | |  |
| **ARI-like** | **Method** | **Variable** | **R** | **L** | | **S** | | | **R** | | **L** | | **S** | **R** | **L** | **S** | **R** | **L** | **S** | **R** | **L** | **S** |  |
| ARI | 4,1 | ARI | 55 | 71 | | 22 | | | 3.47 | | 3.72 | | 2.30 | -0.18 | 0.15 | 0.14 | 3.29 | 3.86 | 2.44 | -3.66 | -3.57 | -2.15 |  |
|  | 12,2 | ARI | 55 | 71 | | 22 | | | 5.14 | | 5.90 | | 3.50 | -0.40 | -0.15 | -0.05 | 4.74 | 5.75 | 3.45 | -5.54 | -6.06 | -3.55 |  |
| ARMA-ARI/ARX | 4,2 | ARI | 55 | 71 | | 22 | | | 4.23 | | 4.45 | | 2.17 | -0.50 | -0.10 | 0.07 | 3.73 | 4.35 | 2.24 | -4.73 | -4.55 | -2.10 |  |
|  | 8,1 | ARI | 55 | 71 | | 22 | | | 14.72 | | 14.64 | | 37.86 | 0.91 | -1.80 | -3.69 | 15.63 | 12.84 | 34.17 | -13.81 | -16.44 | -41.55 |  |
| IR-filter | 11,5 | ARI | 55 | 71 | | 22 | | | 1.08 | | 0.90 | | 0.19 | -0.05 | -0.03 | -0.01 | 1.02 | 0.87 | 0.18 | -1.13 | -0.93 | -0.20 |  |

Results of individual methods per method group. Methods and units are listed in Table 1. T1: measurement 1; T2: measurement 2; bias: T1-T2; INT: interval (=1.96*SD_bias_); ULOA: upper limit of agreement (=mean_bias_-interval); LLOA: lower limit of agreement (=mean_bias_+interval);

**Table S3(c)**. Bland Altman results for correlation-like methods.

|  |  |  |  | |  | |  |  | |  | |  | |  |  |  |  |  |  |  |  |  |  |  |  |  |  |
| --- | --- | --- | --- | --- | --- | --- | --- | --- | --- | --- | --- | --- | --- | --- | --- | --- | --- | --- | --- | --- | --- | --- | --- | --- | --- | --- | --- |
|  |  |  | **nr of cases** | | | | | **LOA** | | | | | | **mean T1 T2** | | | **upper limit** | | | **lower limit** | | |  |  |  |  |  |
| **correlation-like** | **Method** | **Variable** | **R** | **L** | | **S** | | | **R** | | **L** | | **S** | **R** | **L** | **S** | **R** | **L** | **S** | **R** | **L** | **S** |  |  |  |  |  |
| correlation | 5,2 | Cor | 55 | 71 | | 22 | | | 0.30 | | 0.32 | | 0.18 | 0.05 | 0.02 | 0.00 | 0.35 | 0.34 | 0.18 | -0.25 | -0.30 | -0.19 |  |  |  |  |  |
|  | 9,2 | Cor | 55 | 71 | | 22 | | | 0.42 | | 0.43 | | 0.41 | 0.02 | 0.00 | -0.03 | 0.43 | 0.44 | 0.38 | -0.40 | -0.43 | -0.44 |  |  |  |  |  |
|  |  |  |  | |  | |  |  | |  | |  | |  |  |  |  |  |  |  |  |  |  |  |  |  |  |
|  | | | | | | | | | | | | | | | | | | | | | | | |  |  |  |  |

Results of individual methods per method group. Methods and units are listed in Table 1. T1: measurement 1; T2: measurement 2; bias: T1-T2; INT: interval (=1.96*SD_bias_); ULOA: upper limit of agreement (=mean_bias_-interval); LLOA: lower limit of agreement (=mean_bias_+interval);

**Table S4.** Method settings as in Table 1.

| **Method** | **Method group** | **Output Variables** | | | | | | |  |  |  |  |  |  |  |  |  |  | **References** |
| --- | --- | --- | --- | --- | --- | --- | --- | --- | --- | --- | --- | --- | --- | --- | --- | --- | --- | --- | --- |
|  |  | **Gain VLF** | **Gain LF** | **Phase VLF** | **Phase LF** | **Coherence** | **ARI** | **Correlation coefficient** | **Frequency bands** | **Re-sampling** | **Interpolation** | **Detrending** | **Normalization /**  **mean subtraction** | **Filtering** | **Anti-leakage window** | **Window length for analysis (s)** | **Superposition** | **Smoothing** |  |
| 1.1 | 1 | cm/s/mmHg | cm/s/mmHg | rad | rad | au | - | - | 0.02-0.07  0.07-0.20  0.20-0.35 | 1Hz | linear | 3^rd^ order polynomial | - | - | Hanning | 128 | 50% | - | ^1^ |
| 1.2 | 6 | - | - | - | - | - | x | - | - | 5Hz | - | - | N | LPF 1Hz | Hanning | - | - | triangular | ^2^ |
| 2.1 | 2 | cm/s/mmHg | cm/s/mmHg | rad | rad | - | - | - | 0.02-0.07  0.07-0.15  0.15-0.40 | 2Hz | Cubic spline | HPF 0.008 Hz | - | - | Hanning | - | - | - | ^3-6^ |
| 2.2 | 2 | cm/s/mmHg | cm/s/mmHg | rad | rad | - | - | - | 0.02-0.07  0.07-0.15  0.15-0.40 | 2Hz | Cubic spline | HPF 0.008 Hz | - | - | Hanning | - | - | - |  |
| 3.1 | 1 | cm/s/mmHg | cm/s/mmHg | rad | rad | au | - | - | 0.02-0.07  0.07-0.20  0.20-0.35 | - | linear | 3^rd^ order polynomial | - | - | Hanning | 64 | 50% | - | ^1^ |
| 3.2 | 1 | %/% | %/% | - | - | au | - | - | 0.02-0.07  0.07-0.20  0.20-0.35 | - | linear | 3^rd^ order polynomial | - | - | Hanning | 64 | 50% | - |  |
| 4.1 | 6 | - | - | - | - | - | x | - | 0.02-0.07  0.07-0.20  0.20-0.35 | 5Hz | - | - | N | LPF 1Hz | Hanning | 51 | 50%- | triangular | ^2, 7^ |
| 4.2 | 6 | - | - | - | - | - | x | - | - | 1Hz | Spline | - | - | LPF 20Hz | - | 32 | - | - | ^7^ |
| 4.3 | 6 | - | - | - | - | - | x | - | - | 1Hz | Spline | - | - | LPF 20Hz | - | 32 | - | - |  |
| 5.1 | 1 | cm/s/mmHg | cm/s/mmHg | rad | rad | au |  |  | 0.02-0.07  0.07-0.15  0.15-0.40 | - | - | - | - | - | Hanning | 100 | 50% | - | ^1^ |
| 5.2 | 10 | - | - | - | - | - | - | x | - | 1 Hz | - | - | - | - | - | - | - | - | ^8^ |
| 6.1 | 1 | cm/s/mmHg | cm/s/mmHg | rad | rad | au | - | - | 0.02-0.07  0.07-0.15  0.15-0.40 | 0.5Hz | - | Linear | N | - | Hanning | 64 | 50% | - | ^9, 10^ |
| 7.1 | 1 | cm/s/mmHg | cm/s/mmHg | rad | rad | au | - | - | 0.02-0.07  0.07-0.20  0.20-0.35- | 5Hz | Spline | - | N/MS |  | Hanning | 51.2 | 50% | triangular | ^11^ |
| 8.1 | 7 | - | - | - | - | - | x | - | - | 1Hz | Linear | - | N | LPF 0.45Hz | - | 60 | - | - | ^7, 12, 13^ |
| 8.2 | 3 | - | - | rad | rad | - | - | - | - | 1Hz | Cubic spline | HPF 0.005 Hz | - | - | Hanning | 128 | 50% | - | ^14^ |
| 9.1 | 1 | cm/s/mmHg | cm/s/mmHg | rad | rad | au | - | - | 0.02-0.07  0.07-0.15  0.15-0.40 | - | - | - | - | - | Hanning | 100 | 50% | triangular | ^15, 16^ |
| 9.2 | 10 | - | - | - | - | - | - | x | - | - | - | - | - | - | - | - | - | - | ^17^ |
| 11.1 | 1 | cm/s/mmHg | cm/s/mmHg | rad | rad | au | - | - | 0.02-0.07  0.07-0.20  0.20-0.50 | - | Linear | - | - | - | Hanning | 100 | 50% | triangular | ^2^ |
| 11.2 | 1 | %/mmHg | %/mmHg | rad | rad | au | - | - | 0.02-0.07  0.07-0.20  0.20-0.50 | - | Linear | - | - | - | Hanning | 100 | 50% | triangular |  |
| 11.3 | 1 | %/% | %/% | - | - | au | - | - | 0.02-0.07  0.07-0.20  0.20-0.50 | - | Linear | - | - | - | Hanning | 100 | 50% | triangular |  |
| 11.4 | 4 | - | %/% | - | rad | au | - | - | 0.07-0.20 | 1Hz | Linear | Done | N+MS | - | - | - | - | - | ^18^ |
| 11.5 | 9 | - | - | - | - | - | x | - | - | 1Hz | Linear | Done | N | HPF 0.03Hz | - | - | - | - | ^19^ |
| 11.6 | 4 | - | %/% | - | rad | - | - | - | 0.07-0.20 | 1Hz | Linear | Done | N | - | - | - | - | - | ^18^ |
| 12.1 | 1 | cm/s/mmHg | cm/s/mmHg | rad | rad | au | - | - | 0.02-0.07  0.07-0.20  0.20-0.35 | 1Hz | Linear | 3^rd^ order polynomial | - | - | Hanning | 128s | 50% | - | ^1^ |
| 12.2 | 6 | - | - | - | - | - | x | - | - | 5Hz | - | - | N | LPF 1Hz | Hanning | 51.2s | - | triangular | ^2^ |
| 12.3 | 3 | cm/s/mmHg | cm/s/mmHg | rad | rad | - | - | - | - | - | - | - | N | - | - | - | - | - | ^20, 21^ |
| 13.1 | 1 | cm/s/mmHg | cm/s/mmHg | rad | rad | au | - | - | - | 5Hz | - | - | N | LPF 1Hz | Hanning | 51s | 50%- | triangular | ^2, 22^ |
| 14.1 | 5 | cm/s/mmHg | cm/s/mmHg | rad | rad | - | - | - | 0.005-0.04  0.04-0.15  0.15-0.40 | 1Hz | Linear | HPF 0.005Hz | - | LPF | - | 300s | - | - | ^23, 24^ |
| 14.2 | 5 | cm/s/mmHg | cm/s/mmHg | rad | rad | - | - | - | 0.005-0.04  0.04-0.15  0.15-0.40 | 1Hz | Linear | HPF 0.005Hz | - | LPF | - | 300s | - | - |  |
| 14.3 | 2 | cm/s/mmHg | cm/s/mmHg | rad | rad | - | - | - | 0.005-0.04  0.04-0.15  0.15-0.30 | 1Hz | Linear | HPF 0.005Hz | - | LPF | - | - | - | - | ^25, 26^ |
| 14.4 | 2 | cm/s/mmHg | cm/s/mmHg | rad | rad | - | - | - | 0.005-0.04  0.04-0.15  0.15-0.30 | 1Hz | Linear | HPF 0.005Hz | - | LPF | - | - | - | - |  |
| 14.5 | 1 | cm/s/mmHg | cm/s/mmHg | rad | rad | au | - | - | - | - | - | - | - | - | - | - | - | - | ^27^ |

Method: listed in Table 1. Method group: 1=TFA, 2=Laguerre expansions, 3=Wavelets, 4=IR-filter, 5=ARX, 6=ARI, 7=ARMA-ARI/ARX, 9=IR-filter, 10=correlation coefficient; VLF: very low frequency; LF: low frequency; BP: blood pressure; FFT: fast Fourier transform; ARI: autoregulation index;; ARX: autoregressive model with exogenous input; N= normalization; MS=mean subtraction; Parameters of ARI and Correlation coefficient have no units. Centre names are listed in Table 1.

**Table S5 (a).** ICC results surrogate data TFA-like methods

| **Method** | **Gain VLF** | **Gain LF** | **Phase VLF** | **Phase LF** |
| --- | --- | --- | --- | --- |
| 1,1 | 0,84 | 0,86 | 0,47 | 1,00 |
| 2,1 | 0,85 | 0,81 | 0,78 | 0,92 |
| 2,2 | 0,86 | 0,79 | 0,80 | 0,91 |
| 3,1 | 0,76 | 0,87 | 0,32 | 0,98 |
| 3,2 | 0,73 | 0,91 |  |  |
| 5,1 | 0,67 | 0,89 | 0,43 | 0,98 |
| 6,1 | 0,68 | 0,94 | 0,48 | 0,97 |
| 7,1 | 0,76 | 0,87 | 0,67 | 0,98 |
| 9,1 | 0,67 | 0,90 | 0,54 | 0,98 |
| 11,1 | 0,64 | 0,89 | 0,67 | 0,98 |
| 11,2 | 0,60 | 0,93 | 0,72 | 0,99 |
| 11,3 | 0,49 | 0,94 | 0,72 | 0,99 |
| 11,4 |  | 0,49 |  | 0,93 |
| 11,6 |  | 0,42 |  | 0,92 |
| 12,1 | 0,64 | 0,90 | 0,22 | 0,98 |
| 12,3 | 0,55 | 0,36 | 0,29 | 0,79 |
| 13,1 | 0,75 | 0,79 | 0,09 | 0,44 |
| 14,1 | 0,80 | 0,91 | 0,72 | 0,99 |
| 14,2 | 0,78 | 0,91 | 0,52 | 0,99 |
| 14,3 | 0,69 | 0,72 | 0,64 | 0,78 |
| 14,4 | 0,74 | 0,69 | 0,71 | 0,83 |
| 14,5 | 0,63 | 0,88 | 0,22 | 0,98 |

Method: listed in Table 1. TFA: Transfer function analysis; VLF: very low frequency; LF: low frequency;
These results are published in previous research ^28^

**Table S5 (b).** ICC results surrogate data ARI-like and Correlation-like methods

| **Method** | **ARI** | **Correlation** |
| --- | --- | --- |
| 1,2 | 0,94 |  |
| 4,1 | 0,91 |  |
| 4,2 | 0,94 |  |
| 4,3 | 0,90 |  |
| 5,2 |  | 0,35 |
| 8,1 | 0,41 |  |
| 9,2 |  | 0,06 |
| 11,5 | 0,91 |  |
| 12,2 | 0,85 |  |

Method: listed in Table 1. TFA: Transfer function analysis; VLF: very low frequency; LF: low frequency;
These results are published in previous research ^28^

**References**

1. Zhang R, Zuckerman JH, Giller CA, et al. Transfer function analysis of dynamic cerebral autoregulation in humans. *Am J Physiol* 1998; 274: H233-241.

2. Panerai RB, White RP, Markus HS, et al. Grading of cerebral dynamic autoregulation from spontaneous fluctuations in arterial blood pressure. *Stroke* 1998; 29: 2341-2346.

3. Marmarelis VZ. *Nonlinear Dynamic Modeling of Physiological Systems*. New Jersey: Wiley-Interscience, 2004.

4. Marmarelis VZ, Shin DC, Orme M, et al. Time-varying modeling of cerebral hemodynamics. *IEEE Trans Biomed Eng* 2014; 61: 694-704. DOI: 10.1109/TBME.2013.2287120.

5. Marmarelis VZ, Shin DC, Orme ME, et al. Model-based quantification of cerebral hemodynamics as a physiomarker for Alzheimer's disease? *Ann Biomed Eng* 2013; 41: 2296-2317. DOI: 10.1007/s10439-013-0837-z.

6. Marmarelis VZ, Shin DC, Orme ME, et al. Model-based physiomarkers of cerebral hemodynamics in patients with mild cognitive impairment. *Med Eng Phys* 2014; 36: 628-637. DOI: 10.1016/j.medengphy.2014.02.025.

7. Panerai RB, Eames PJ and Potter JF. Variability of time-domain indices of dynamic cerebral autoregulation. *Physiol Meas* 2003; 24: 367-381.

8. Caicedo A, Varon C, Hunyadi B, et al. Decomposition of Near-Infrared Spectroscopy Signals Using Oblique Subspace Projections: Applications in Brain Hemodynamic Monitoring. *Front Physiol* 2016; 7: 515. DOI: 10.3389/fphys.2016.00515.

9. Muller M, Bianchi O, Erulku S, et al. Changes in linear dynamics of cerebrovascular system after severe traumatic brain injury. *Stroke* 2003; 34: 1197-1202. DOI: 10.1161/01.STR.0000068409.81859.C5.

10. Muller MW and Osterreich M. A comparison of dynamic cerebral autoregulation across changes in cerebral blood flow velocity for 200 s. *Front Physiol* 2014; 5: 327. DOI: 10.3389/fphys.2014.00327.

11. Gommer ED, Shijaku E, Mess WH, et al. Dynamic cerebral autoregulation: different signal processing methods without influence on results and reproducibility. *Med Biol Eng Comput* 2010; 48: 1243-1250. DOI: 10.1007/s11517-010-0706-y.

12. Liu Y and Allen R. Analysis of dynamic cerebral autoregulation using an ARX model based on arterial blood pressure and middle cerebral artery velocity simulation. *Med Biol Eng Comput* 2002; 40: 600-605.

13. Liu Y, Birch AA and Allen R. Dynamic cerebral autoregulation assessment using an ARX model: comparative study using step response and phase shift analysis. *Med Eng Phys* 2003; 25: 647-653.

14. Peng T, Rowley AB, Ainslie PN, et al. Wavelet phase synchronization analysis of cerebral blood flow autoregulation. *IEEE Trans Biomed Eng* 2010; 57: 960-968. DOI: 10.1109/TBME.2009.2024265.

15. van Beek AH, Lagro J, Olde-Rikkert MG, et al. Oscillations in cerebral blood flow and cortical oxygenation in Alzheimer's disease. *Neurobiology of aging* 2012; 33: 428 e421-431. DOI: 10.1016/j.neurobiolaging.2010.11.016.

16. van Beek AH, Olde Rikkert MG, Pasman JW, et al. Dynamic cerebral autoregulation in the old using a repeated sit-stand maneuver. *Ultrasound Med Biol* 2010; 36: 192-201. DOI: 10.1016/j.ultrasmedbio.2009.10.011.

17. Heskamp L, Meel-van den Abeelen A, Katsogridakis E, et al. Convergent cross mapping: a promising technique for future cerebral autoregulation estimation. *Cerebrovasc Dis* 2013; 35: 15-16.

18. Panerai RB, Simpson DM, Deverson ST, et al. Multivariate dynamic analysis of cerebral blood flow regulation in humans. *IEEE Trans Biomed Eng* 2000; 47: 419-423. DOI: 10.1109/10.827312.

19. Simpson DM, Panerai RB, Evans DH, et al. A parametric approach to measuring cerebral blood flow autoregulation from spontaneous variations in blood pressure. *Ann Biomed Eng* 2001; 29: 18-25.

20. Grinsted A, Moore JC and Jevrejeva S. Application of the cross wavelet transform and wavelet coherence to geophysical time series. *Nonlinear Proc Geoph* 2004; 11: 561-566.

21. Torrence C and Webster PJ. Interdecadal changes in the ENSO-monsoon system. *J Climate* 1999; 12: 2679-2690. DOI: Doi 10.1175/1520-0442(1999)012<2679:Icitem>2.0.Co;2.

22. Panerai RB, Rennie JM, Kelsall AW, et al. Frequency-domain analysis of cerebral autoregulation from spontaneous fluctuations in arterial blood pressure. *Med Biol Eng Comput* 1998; 36: 315-322.

23. Mitsis GD, Zhang R, Levine BD, et al. Modeling of nonlinear physiological systems with fast and slow dynamics. II. Application to cerebral autoregulation. *Ann Biomed Eng* 2002; 30: 555-565.

24. Mitsis GD, Zhang R, Levine BD, et al. Autonomic neural control of cerebral hemodynamics. *IEEE Eng Med Biol Mag* 2009; 28: 54-62. DOI: 10.1109/MEMB.2009.934908.

25. Mitsis GD, Poulin MJ, Robbins PA, et al. Nonlinear modeling of the dynamic effects of arterial pressure and CO2 variations on cerebral blood flow in healthy humans. *IEEE Trans Biomed Eng* 2004; 51: 1932-1943. DOI: 10.1109/TBME.2004.834272.

26. Kostoglou K, Debert CT, Poulin MJ, et al. Nonstationary multivariate modeling of cerebral autoregulation during hypercapnia. *Med Eng Phys* 2014; 36: 592-600. DOI: 10.1016/j.medengphy.2013.10.011.

27. Meel-van den Abeelen AS, Simpson DM, Wang LJ, et al. Between-centre variability in transfer function analysis, a widely used method for linear quantification of the dynamic pressure-flow relation: the CARNet study. *Med Eng Phys* 2014; 36: 620-627. DOI: 10.1016/j.medengphy.2014.02.002.

28. Sanders ML, Claassen J, Aries M, et al. Reproducibility of dynamic cerebral autoregulation parameters: a multi-centre, multi-method study. *Physiol Meas* 2018; 39: 125002. 2018/12/14. DOI: 10.1088/1361-6579/aae9fd.
